# Caprine Bactenecins as Promising Tools for Developing New Antimicrobial and Antitumor Drugs

**DOI:** 10.3389/fcimb.2020.552905

**Published:** 2020-10-19

**Authors:** Pavel M. Kopeikin, Maria S. Zharkova, Alexander A. Kolobov, Maria P. Smirnova, Maria S. Sukhareva, Ekaterina S. Umnyakova, Vladimir N. Kokryakov, Dmitriy S. Orlov, Boris L. Milman, Sergey V. Balandin, Pavel V. Panteleev, Tatiana V. Ovchinnikova, Aleksey S. Komlev, Alessandro Tossi, Olga V. Shamova

**Affiliations:** ^1^ Laboratory of Design and Synthesis of Biologically Active Peptides, Department of General Pathology and Pathophysiology, FSBSI Institute of Experimental Medicine, Saint Petersburg, Russia; ^2^ Laboratory of Peptide Chemistry, State Research Institute of Highly Pure Biopreparations, Saint Petersburg, Russia; ^3^ Science-Educational Center, M.M. Shemyakin & Yu.A. Ovchinnikov Institute of Bioorganic Chemistry, The Russian Academy of Sciences, Moscow, Russia; ^4^ Department of Biotechnology, I.M. Sechenov First Moscow State Medical University, Moscow, Russia; ^5^ Department of Life Sciences, University of Trieste, Trieste, Italy

**Keywords:** proline-rich antimicrobial peptides, bactenecins, structure–activity relationship, antibacterial activity, antibiofilm activity, antitumor activity, synergy, antibiotics

## Abstract

Proline-rich antimicrobial peptides (PR-AMPs) having a potent antimicrobial activity predominantly toward Gram-negative bacteria and negligible toxicity toward host cells, are attracting attention as new templates for developing antibiotic drugs. We have previously isolated and characterized several bactenecins that are promising in this respect, from the leukocytes of the domestic goat *Capra hircus*: ChBac5, miniChBac7.5N-α, and -β, as well as ChBac3.4. Unlike the others, ChBac3.4 shows a somewhat unusual pattern of activities for a mammalian PR-AMP: it is more active against bacterial membranes as well as tumor and, to the lesser extent, normal cells. Here we describe a SAR study of ChBac3.4 (RFRLPFRRPPIRIHPPPFYPPFRRFL-NH2) which elucidates its peculiarities and evaluates its potential as a lead for antimicrobial or anticancer drugs based on this peptide. A set of designed structural analogues of ChBac3.4 was explored for antibacterial activity toward drug-resistant clinical isolates and antitumor properties. The *N*-terminal region was found to be important for the antimicrobial action, but not responsible for the toxicity toward mammalian cells. A shortened variant with the best selectivity index toward bacteria demonstrated a pronounced synergy in combination with antibiotics against Gram-negative strains, albeit with a somewhat reduced ability to inhibit biofilm formation compared to native peptide. *C*-terminal amidation was examined for some analogues, which did not affect antimicrobial activity, but somewhat altered the cytotoxicity toward host cells. Interestingly, non-amidated peptides showed a slight delay in their impact on bacterial membrane integrity. Peptides with enhanced hydrophobicity showed increased toxicity, but in most cases their selectivity toward tumor cells also improved. While most analogues lacked hemolytic properties, a ChBac3.4 variant with two additional tryptophan residues demonstrated an appreciable activity toward human erythrocytes. The variant demonstrating the best tumor/nontumor cell selectivity was found to more actively initiate apoptosis in target cells, though its action was slower than that of the native ChBac3.4. Its antitumor effectiveness was successfully verified *in vivo* in a murine Ehrlich ascites carcinoma model. The obtained results demonstrate the potential of structural modification to manage caprine bactenecins’ selectivity and activity spectrum and confirm that they are promising prototypes for antimicrobial and anticancer drugs design.

## Introduction

Proline-rich antimicrobial peptides (PR-AMPs) comprise a distinct group of AMPs, which contain 20%–50% of proline residues in their amino acid sequence, and generally have a high positive net charge, normally due to the presence of numerous arginine residues. These peptides are predominantly highly active against Gram-negative bacteria, whereas their toxicity toward host cells is often negligible. They are therefore considered promising templates for development of novel potential antibiotic. The most well-studied PR-AMPs are possibly those from insects, where they represent a predominant class of AMPs providing anti-infective protection for these animals (Otvos, 2000; Bulet, 2008). At the moment, some derivatives of insect PR-AMPs are showing considerable effectiveness in preclinical animal studies (Schmidt et al., 2016; Schmidt et al., 2017). In mammals PR-AMPs have been found in leukocytes of artiodactyl animals and their phylogenetic descendants: cattle (Gennaro et al., 1989), sheep (Huttner et al., 1998; Shamova et al., 1999; Anderson and Yu, 2003), deer (Treffers et al., 2005), pigs (Agerberth et al., 1991; Harwig et al., 1995), dolphins (Mardirossian et al., 2018a), etc. Mammalian PR-AMPs also possess a marked antimicrobial activity against Gram-negative bacteria including strains causing severe nosocomial infections and have a good prospect for clinical application (Vitali, 2015; Dolzani et al., 2019).

Pro-rich sequences are known to be frequent participants of various protein–protein interactions; varied protein domains are reported to require certain proline-including patterns in their ligands (Kay et al., 2000; Srinivasan and Dunker, 2012). Thus, it is not overly surprising, that PR-AMPs demonstrate a special mode of antimicrobial action different from the many other AMPs of innate immunity. Due to the possibility of specific proline-mediated protein–protein interactions, PR-AMPs implement their antimicrobial action through several intracellular molecular targets, without causing significant damage to bacterial membranes, unlike most other structural classes of AMPs. They can bind to the chaperone DnaK, as was shown for insect pyrrochoricin, bovine Bac7 and ovine OaBac7.5 fragments, and modulate its ATPase activity, disturbing the protein folding process in the cell (Kragol et al., 2001; Scocchi et al., 2009; Zahn et al., 2013; Zahn et al., 2014). They also interact with the 70S ribosome, impairing the translation process, as reported for apidaecins, oncocins, the bovine Bac7 fragment 1–35 (Krizsan et al., 2014; Mardirossian et al., 2014; Krizsan et al., 2015; Roy et al., 2015) and Bac5 fragments (Mardirossian et al., 2018b).

The main subjects of the present study are PR-AMPs from the domestic goat *Capra hircus*, in particular bactenecin ChBac3.4. Previously we have isolated several proline-rich bactenecins from the goat leukocytes, including caprine bactenecin 5 (ChBac5), bactenecin 3.4 (ChBac3. 4), and minibactenecins (mini-ChBac7.5Nα and β—two naturally processed active *N*-terminal fragments of caprine Bac7.5) (Shamova et al., 1999; Shamova et al., 2009; Shamova et al., 2016). The names are derived from ***C*** *apra* ***h*** *ircus* and peptide size. We have shown that chemically synthesized ChBac3.4, ChBac5, mini-ChBac7.5Nα, mini-ChBac7.5Nβ exhibit a pronounced *in vitro* antimicrobial activity against drug-resistant clinical isolates (*Escherichia coli*, *Acinetobacter baumannii*, *Pseudomonas aeruginosa*, *Klebsiella pneumoniae*) (Shamova et al., 2017; Zharkova et al., 2018).

ChBac5 and minibactenecins demonstrate a spectrum of activity and mode of action that is typical for PR-AMPs, including their bovine orthologs (Bac5 and Bac7) (Skerlavaj et al., 1996; Shamova et al., 2009; Shamova et al., 2016), whereas another caprine bactenecin—ChBac3.4—noticeably differs from the majority of the representatives of this peptides family by having a wider spectrum of antimicrobial action, a more pronounced effect in damaging microbial membranes, and a cytotoxic effect against mammalian cells which is uncommon amongst PR-AMPs (Shamova et al., 2009). Elucidation of the structural basis of such differences between ChBac3.4 and the other PR-AMPs was of interest, both for understanding the general principles of PR-AMPs action on bacteria and for developing new antibiotics structurally based on natural peptides.

The amino acid sequence of ChBac3.4 is following: **RFR**L**P**F**RRPPI R**IHPPPFYPPFRRFL-NH_2_, with a significant sequence similarity (66% identity) particularly in the *N*-stretch (in bold) of ChBac5 (**RFR**P**P**I**RRPPI** **R** PPF NPPF RPPV RPPF RPPF RPPF RPPI GPFP-NH_2_), and the same *C*-terminal amidation signal (GRR). However ChBac5 includes eight repeated RPPX or NPPX motifs (underlined, where X is a hydrophobic residue—F, I, V) whereas ChBac3.4 has only one. The region after residue 12 of ChBac3.4 molecule, where the histidine residue is situated, aligns with the *C*-terminal fragment of ChBac5 with only 35% identity (increasing to 55% if the GRR amidation signal is considered).

To determine which structural features of bactenecin ChBac3.4 are crucial for antibacterial activity, as well as for manifestation of its toxic properties toward host cells, we have designed and synthesized a set of structural analogues of this peptide (**Table 1**) and explored their antibacterial potency against drug-resistant clinical isolates and cytotoxicity toward normal and tumor-derived mammalian cells. By comparing the effects of the designed variants against bacterial and eukaryotic cells, we could also determine which of the bactenecin modifications confer improved characteristics for the practical use.

**Table 1:** Primary structure of designed analogues of the natural bactenecin ChBac3.4.

Abbreviation	Amino acid sequence^a^	Description	Net charge^b^
*original peptide*			
ChBac3.4-NH_2_	** RFRLPFRRP PIRIHPPPFYP PFRRFL-NH_2_**	amino acid sequence is identical to that of natural ChBac3.4; *C*-terminal amidated	+8
*full-lenght variants*			
ChBac3.4-COOH	** RFRLPFRRP PIRIHPPPFYP PFRRFL-COOH**	a non-amidated at the *C*-terminus analogue of ChBac3.4	+7
ChBac3.4-1-NH_2_	** RFRLPFRRIHPPPFVRIHPPPFYRRFL-NH_2_**	contains an additional copy of a region 12–18 (RIHPPPF), differing the most from ChBac5 sequence with its RPPX-motif’s repeats, where X = I, F, V	+8
ChBac3.4-1-COOH	** RFRLPFRRIHPPPFVRIHPPPFYRRFL-COOH**	a non-amidated at the *C*-terminus analogue of ChBac3.4-1	+7
RFR-ChBac3.4-1-NH_2_	**RFRRFRLPFRRIHPPPFVRIHPPPFYRRFL-NH_2_**	ChBac3.4-1variant with additional RFR triplet at the *N*-terminus; *C*-terminal amidated	+10
ChBac3.4-2-COOH	** RFRLPFRRPWPIRIHPPPFYPWPFRRFL-COOH**	ChBac3.4 variant with two inserts of tryptophan residues at positions 10 and 22; non-amidated at the *C*-terminus	+7
ChBac3.4(Н-)	** RFRLPFRRP PIRIPPPFYP PFRRFL-NH_2_**	ChBac3.4 analogue with a deletion of histidine residue at position 14; *C*-terminal amidated	+8
*truncated variants*			
ChBac3.4 (12-26)	** RIHPPPFYP PFRRFL-NH_2_**	*C*-terminal fragment of ChBac3.4 from 12st to 18th amino acid residue; *C*-terminal amidated	+4
ChBac3.4(1-19)-NH_2_	** RFRLPFRRP PIRIHPPPFY-NH_2_**	*N*-terminal fragment of ChBac3.4 from 1st to 19th amino acid residue; *C*-terminal amidated	+6
ChBac3.4(1-14)-NH_2_	** RFRLPFRRP PIRIH-NH_2_**	*N*-terminal fragment of ChBac3.4 from 1st to 14th amino acid residue; *C*-terminal amidated	+6
RFR-ChBac3.4 (1-14)-NH_2_	**RFRRFRLPFRRP PIRIH-NH_2_**	ChBac3.4(1-14) variant with additional RFR triplet at the *N*-terminus; *C*-terminal amidated	+8

a The C-terminal 12–18 motif present in ChBac3.4, but not in ChBac5 is underlined, and the unique His residue is indicated in red. The RFP motif added to the N-terminal of some variants is in green; Trp residues inserted into ChBac3.4-2-COOH variant are marked blue.

b The charge is considered at pH 7.4, with His being neutral, and takes amidation into account.

The multitargeted but specific mechanisms of action of PR-AMPs render their structure–activity relationship (SAR) studies more complicated and less predictable compared to AMPs that have a mainly membranolytic activity, as it is not possible to use just a general approach based on variation of the physico-chemical properties of residues in the peptides molecule, but the possible effects of the presence of certain single amino acids or residue motifs and their positioning must be taken into consideration given their role in PR-AMP–target protein interactions that may be involved in their action. In other words, alongside general dependencies, in our ChBac3.4 variants design (**Table 1**) we wanted to clarify a number of specific aspects:

– A first aspect was the role of *C*-terminal amidation in the PR-AMP’s effects on bacteria or host cells; modifications differing only by its presence or absence were thus included for selected variants (indicated by -NH_2_ or -COOH after the peptide name).
– Secondly, the importance of the *N*-terminal region was assessed for various types of activity. For some mammalian PR-AMPs, this region is found to be crucial for antimicrobial activity (Shi et al., 1996; Skerlavaj et al., 1999; Tokunaga et al., 2001). However, the significance of this region for the manifestation of toxic effects toward cells of higher eukaryotes has not been studied. In this respect, the activity of both *C*- and *N*-terminally truncated versions of ChBac3.4 was examined.
– Thirdly, we considered the role of the histidine residue (considering also that it is not encountered in other goat bactenecins) as this amino acid is known for its versatile involvement in mediating protein interactions (Liao et al., 2013). It was either omitted from the RIHPPPF motif in the ChBac3.4 sequence [ChBac3.4(Н-)] or a second RIHPPPF motif was added [ChBac3.4-1 series]. This also allowed us to investigate the role of the region most dissimilar to ChBac5 (between residues 12 and 18 in ChBac3.4) as a whole in determining some of the unusual activity pattern of the PR-AMP.
– Based on the published data on the importance of the *N*-terminal sequence in some other bactenecins, and with a view to enhancing said activity (Gennaro et al., 2002), an additional RFR motif was added either to a variant lacking the full RIHPPPF motif [RFR-ChBac3.4(1-14)-NH2] or to a variant with a double such repeat [RFR-ChBac3.4-1-NH_2_].
– Finally, we also explored the effect of introducing tryptophan residues into the ChBac3.4 sequence to improve its antibacterial properties, as another specific group of AMPs rich in this amino acid is known for a broad activity spectrum, though quite frequently accompanied by considerable hemolycity.

**Figure 1:**
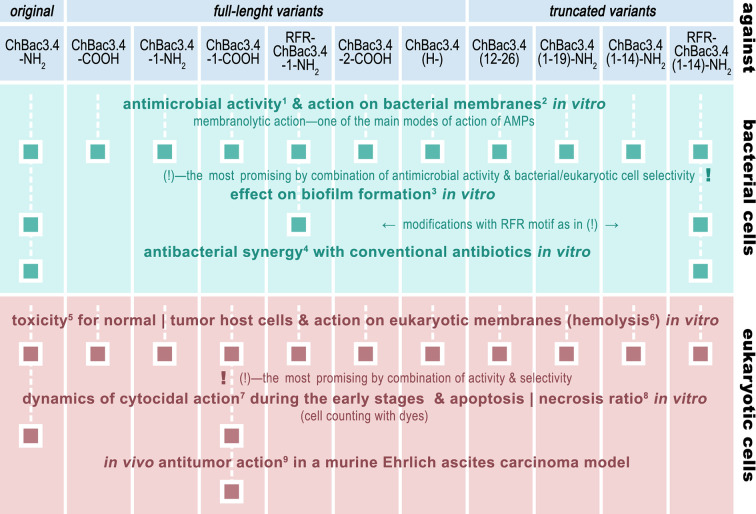
The roadmap of ChBac3.4 modifications’ activity testing performed in this study. The methods used were: 1) broth microdilution assay; 2) spectrophotometric membrane permeabilization assay on *E. coli* ML-35p; 3) crystal violet assay; 4) checkerboard titration; 5) MTT-test; 6) hemolysis assay; 7) light microscopy & cell counting with Trypan blue dye; 8) fluorescent microscopy & cell counting with Annexin V-Cy3 Apoptosis Detection kit dyes; 9) Kaplan–Meier survival curves analysis.

Given the set of ChBac3.4 variants listed in **Table 1**, it was first necessary to determine the effect of the sequence variations on the antimicrobial activity and toxicity spectra, also taking into account the capacity to interact with bacterial and eukaryotic membranes. Variants showing an increased effectiveness combined with an improved selectivity of action were further investigated by testing their impact against *i)* the formation of biofilms, *ii)* their ability to synergize with conventional antibiotics, and *iii)* their antitumor potential not only *in vitro*, but also *in vivo*—in a murine Ehrlich carcinoma model (**Figure 1**).

## Materials and Methods

### Antimicrobial Peptides

Antimicrobial peptides were produced *via* the Fmoc solid phase synthetic approach on a Liberty microwave peptide synthesizer (CEM Corp., USA) or a Symphony X (Protein Technologies, USA), using standard synthesis protocols. After chain assembly, linear unprotected peptide was cleaved from the polymer using a TFA cleavage cocktail (TFA/triisopropylsilane/water/ethandithiol = 94/1/2.5/2.5) and purified by semi-preparative RP-HPLC on a Gilson chromatograph using a Waters SymmetryPrep C18 column, 9×300 mm, 100Å, 7µm, or on a Beckman Gold System chromatograph using a Vydac C18 column, 10×250 mm, 5 µm. According to analytical RP-HPLC data (Luna C18 column, 4.6×250mm, 100Å, 5µm) the purity of the bactenecin and its analogues was 96-98%. The peptides have been characterized by MALDI-TOF mass spectrometry (see the **Supplementary Material**) Alpha-cyano-4-hydroxycinnamic acid was used as a matrix in a 1:2 mixture of acetonitrile and a 0.1% aqueous solution of TFA. Mass spectra were obtained on an Ultraflextreme MALDI TOF/TOF instrument (Bruker Daltonics, Germany) in the positive ion mode and recorded under the following conditions: UV laser with a frequency of 1000 Hz, power of 20%–30% and 1000–5000 laser pulses, reflection mode. A standard membrane-active peptide protegrin 1 (PG-1) was kindly provided by Prof. R. Lehrer (University of California, Los Angeles). The peptide ChBac3.4-1-COOH was alternatively produced using recombinant expression system by means of the following procedure. The recombinant plasmid for expression of the non-amidated peptide was constructed with the use of pET-based vector as described previously (Panteleev and Ovchinnikova, 2017). The expression cassette included the T7 promoter, ribosome binding site (RBS), and the sequence encoding the recombinant protein that included an His-tag, the carrier protein (*E. coli* thioredoxin A[M37L]), a methionine residue, and a target peptide. *E. coli* BL21(DE3) cells transformed with the corresponding plasmid were grown at 37°C in Lysogeny broth (LB) medium supplemented with 100 μg/ml ampicillin, 20 mM glucose, and 1 mM magnesium sulfate, and were induced at OD_600_ ~0.7–1.0 with 0.2 mM isopropyl β-D-1-thiogalactopyranoside (IPTG) for 6 h at 30°C and 220 rpm. After centrifugation the pelleted cells were suspended and sonicated in the 100 mM phosphate buffer (pH 7.8) containing 20 mM imidazole and 6 M guanidine hydrochloride to fully solubilize the fusion protein. Purification of the peptide involved immobilized metal affinity chromatography (IMAC) of cell lysate with the use of Ni Sepharose (GE Healthcare), CNBr cleavage of the fusion protein, and RP-HPLC with the use of Reprosil-pur C18-AQ column (Dr. Maisch GmbH) as described previously (Panteleev et al., 2018). The expression level of the recombinant protein as well as the effectiveness of the purification was monitored using Tricine-sodium dodecyl sulfate polyacrylamide gel electrophoresis (Tricine-SDS-PAGE). The obtained peptide was analyzed by MALDI-TOF mass-spectrometry using Reflex III instrument (Bruker Daltonics) and then was lyophilized (for chromatograms and mass spectra see **Supplementary Data**).

### Antibacterial Assays

Bacterial strains used were as follows:

– Laboratory strains *Escherichia coli* ML-35р and MRSA (methicillin-resistant *Staphylococcus aureus*) ATCC 33591 were kindly provided by Professor R. Lehrer, University of California, Los Angeles, USA; and *Staphylococcus aureus* SG-511 by Professor H-G Sahl, University of Bonn.
– Drug-resistant bacterial strains *Acinetobacter baumannii* 7226/16 (resistant to imipenem, gentamicin, tobramycin, ciprofloxacin, trimethoprim/sulfamethoxazole), *Pseudomonas aeruginosa* MDR 522/17 (resistant to meropenem, ceftazidime, cefixime, amikacin, gentamicin, netilmicin, ciprofloxacin, colistin), *Klebsiella pneumoniae* ESBL 344/17 (resistant to ampicillin and other penicillins), and *Staphylococcus aureus* 1399/17 (resistant to ampicillin, oxacillin, gentamicin, amikacin, ofloxacin) were isolated from infected wounds, *Escherichia coli* ESBL 521/17 (resistant to ampicillin, amoxicillin/clavulonic acid, cefotaxime, ceftazidime, cefixime, aztreonam, netilmicin, ciprofloxacin, trimethoprim/sulfamethoxazole) from the urine of patients (see **Supplementary Table 1**). These clinical isolates were generously supplied by Prof. G.E. Afinogenov from Saint Petersburg State University and Dr. A. Afinogenova from the Research Institute of Epidemiology and Microbiology named after L. Pasteur, Saint Petersburg, Russia; initial susceptibility testing was performed by their colleagues from said institutions.

#### Broth Microdilution Assay

The minimal inhibitory concentrations (MIC) of peptides were determined using microdilution assay in Müller–Hinton (MH) broth in general accordance with the guidelines of the European Committee for Antimicrobial Susceptibility Testing. Test plates were additionally pretreated with 0.1% bovine serum albumin (BSA) in distilled H_2_O (incubated for 1 h at 37°C) to reduce possible non-specific binding of peptides to the plastic surface of the wells, as proposed for AMP activity measuring (Tossi et al., 1997; Wiegand et al., 2008). Two-fold serial dilutions of peptides starting with 2 × stock concentration were prepared in sterile 10 mM sodium phosphate (Na-P) buffer, pH 7.4, with 0.1% BSA. Thus, final solutions contained 50% of Na-P diluted peptide and 50% of MH broth with bacteria. Bacteria for testing were grown overnight, then transferred into a fresh portion of 2.1% MH medium and additionally incubated for 2–3 h to obtain bacterial culture in its mid-logarithmic growth phase, which was diluted down to the final concentration of 1 × 10^6^ CFU/ml. Concentration of bacteria in growth medium was calculated based on its absorbance at 620 nm.

After 20 h of incubation of AMPs with bacteria MICs were determined as lowest concentrations of AMPs completely preventing the visible growth of bacteria. Tests were performed in 60-well Terasaki microplates (10 µl end volume, V-shaped bottom; Sarstedt, Germany); dilutions in each test were made in triplicate. Final MIC values were calculated as medians based on the results of 3–5 independent experiments.

#### Combined Antimicrobial Activity Assessment by Checkerboard Titration

The combined antimicrobial action of AMPs and antibiotics was analyzed under the same conditions as in broth microdilution assay, using the checkerboard titration template for diluting the tested components. One compound (A) was diluted two-fold every row of the test plate, and the other compound (B) was similarly diluted every column, creating the variety of different combinations of their concentrations. The first column and the last row contained only serial dilutions of one component to assess their exact MICs during the experiment. After the overnight incubation with bacteria Fractional Inhibitory Concentration Indices (FICI) were found as FIC Index = [A]/[MIC A] + [B]/[MIC B], where [A] and [B] are fractional concentrations of A and B in combination effectively inhibiting bacterial growth, and [MIC A] and [MIC B] are MICs of A or B used alone. Combined effect was classified based on the minimal value of FICI for the tested combination: minFICI ≤ 0.5 indicates synergy; 0.5 < minFICI ≤ 1 indicates additivity (the effects of substances simply add without the amplification seen in synergy); 1 < minFICI ≤ 2 indicates independent action (one substance acts as if the other is not present); minFICI > 2 indicates antagonism, due to two-fold serial dilution error margin (Hsieh et al., 1993; Orhan et al., 2005; Fehri et al., 2007).

#### Membrane Permeabilization Assay

The effects of peptides on the permeability of *E.coli* ML-35p inner and outer membranes to chromogenic reporter molecules were assessed spectrophotometrically, using a method initially designed by Prof. Lehrer and coauthors (Lehrer et al., 1988), which relies on specific features of the *E. coli* ML-35p strain. This bacterium expresses β-lactamase in its periplasmic space, and due to a modified lactose operon has no permeases to transport β-galactosides through its inner membrane, while constitutively expressing β-galactosidase in its cytoplasm. Nitrocefin [3-(2,4-dinitrostyryl)-(6R,7R)-7-(2-thienyl acetamido)ceph-3-em-4-carboxylic acid, chromogenic β-lactamase substrate, Calbiochem-Novabiochem, San-Diego, USA] and *o*-nitrophenyl-β-D-galactoside (ONPG, β-galactosidase substrate, Sigma, La Jolla, USA) were used to monitor the changes in the permeability of the bacterial outer and inner membranes, respectively. The pink-colored product of nitrocefin hydrolysis was detected at 486 nm, and yellow-colored *o*-nitrophenyl at 420 nm.

Bacteria used for this assay were grown overnight at 37°C in the sterile 3% Trypticase soy broth (TSB), washed trice with 10 mM Na-P buffer, pH 7.4, diluted down to 1 × 10^8^ CFU/ml (so that the optical density (OD) of bacterial culture was 0.4 at 620 nm), and immediately used. Experiments were conducted in transparent flat-bottomed 96-well microplates, test wells contained peptides in concentration of 2×MIC or the equivalent volume of acidified water (for controlling passive substrate degradation), 20 μM of nitrocefin or 2.5 mM of ONPG, 10 mM Na-P buffer, pH 7.4, with 100 mM NaCl and 2.5 × 10^7^ CFU/ml of washed bacteria; the final volume in each well was 100 μl. OD of the mixtures was recorded once per minute for 1 h with SpectraMax 250 Microplate Spectrophotometer (Molecular Devices, Sunnyvale, USA) using its manufacturer-supplied software SOFTmax PRO. Measurement was started right after adding the bacteria and was run at 37°C with shaking the plate for 5 s before each read. Data were plotted using Sigma Plot 11; typical experimental curves are presented at the corresponding Figure.

#### Biofilm Formation Assessment by the Crystal Violet Assay

Quantification of the biofilms forming in the presence of various concentrations of tested AMPs was performed using the crystal violet assay according to general guidelines (Merritt et al., 2006). Tests were performed in polystyrene 96-well plates with U-shaped bottom. Peptides were serially diluted in a bacterial growth medium (MH for *A. baumannii* or TSB for *P. aeruguinosa*) in a volume of 50 μl per well. Overnight cultures of tested bacteria in a stationary phase of growth were 50 times diluted and introduced into the experimental wells at the same volume of 50 μl. Samples were incubated for 24 h at 37°C. The content of the wells was then shacked out, the plates were gently washed from unattached planktonic bacteria in still water (poured into a large enough vessel); bacterial cells and matrix components adhered to the walls of the wells were stained with a 0.1% aqueous solution of crystal violet dye: 125 μl of dye solution was put into each well and incubated at room temperature for 10 min. After staining dye solution was removed, the plates were washed with clean water and allowed to air-dry. Finally, the bound dye was redissolved by adding 200 μl of 30% acetic acid into each well, incubating it for 15 min at room temperature, and then thoroughly mixing the content of the wells by pipetting. One hundred and twenty five microliters of crystal violet extract from each well were transferred into a flat-bottomed microtiter plate, and the optical density was measured at a wavelength of 560–595 nm (depending on the maximum of absorption in a particular experiment). Experimental samples were made in quadruplicates, and there were 8–9 repeats of control samples without peptides in each test. Presented results are medians calculated based on 3 independent experiments.

### Toxicity Testing Against Eukaryotic Cells

#### Hemolysis Assay

Hemolytic activity of peptides toward human erythrocytes was determined using a standard protocol (Tossi et al., 1997). Peripheral blood of healthy volunteers was collected into EDTA-coated vacutainer tubes and then repeatedly washed with phosphate-buffered saline (PBS), precooled to 4°C, to remove any trace of plasma components and of anticoagulant. The washing cycle included centrifuging the samples at 300 g at 4°C for 10 min, removing the supernatant and resuspending the cells in a fresh aliquot of PBS. After the third round, 280 μl of cellular precipitate were resuspended with PBS up to 10 ml to obtain a suspension of stock red blood. 27 μl of this stock were mixed with 3 μl of the tested peptides, also diluted in PBS to various concentrations; the end concentration of erythrocytes was 2.5% v/v. Mixtures were incubated for 30 min at 37°C and then centrifuged for 3 min at 10,000 g. Hemoglobin release from the lysed erythrocytes was spectrophotometrically measured in the supernatants at 540 nm, using a SpectraMax 250 Spectrophotometer (Molecular Devices, USA). The percentage of hemolysis in test samples was calculated by comparison to a positive control for total hemolysis (100% lysis) where 3 μl of 1% v/v Triton X-100 was used instead of the peptides, and with a negative control (0% lysis) where only PBS was added to erythrocytes, according to the following formula:

(1) $$Hemolysis(\%)=\frac{\left(OD_{sample}-OD_{0\%\;lysis}\right)}{\left(OD_{100\%\;lysis}-OD_{0\%\;lysis}\right)}\times \;100\%,$$

where OD_sample_, OD_0% lysis_, and OD_100% lysis_ are respectively the optical density values at 540 nm for the test sample and the negative and positive controls.

Experiments were repeated three times and in each case in triplicate (samples and controls). Tests were carried out in accordance with the Declaration of Helsinki, written informed consent was given by all donors beforehand.

#### MTT Test

Cytotoxic activity of peptides toward various types of eukaryotic cells was assessed using the standard MTT [3-(4,5-Dimethylthiazol-2-yl)-2,5-diphenyltetrazolium bromide] test (Mosmann, 1983). Human peripheral blood mononuclear cells (PBMC) were isolated from the peripheral blood of healthy donors. Whole heparinized blood diluted 3-fold with sterile PBS was carefully layered upon the 5 ml of the sterile Ficoll-400 (Pharmacia, Sweden) with a density of 1.077 in a sterile 50 ml conical tube and then centrifuged at 600 g and 4°C for 40 min. The mononuclear “ring” remaining above the Ficoll was collected using a transfer pipette and placed in another tube. Cells were washed twice with sterile PBS, resuspended in RPMI-1640 culture medium and immediately used for the test. Tumor cell lines were purchased from Biolot (Saint Petersburg, Russia) and cultured at 37°C and 5% CO_2_ in RPMI-1640 medium (Biolot, Saint Petersburg, Russia) supplemented with 10% fetal bovine serum, glutamine, and penicillin-streptomycin combination. For the experiments, cells were washed from the medium they were growing in and resuspended in serum- and antibiotic-free RPMI-1640. Cells were added at not less than 2 × 10^4^ per well in sterile flat bottom 96-well microplates containing serial dilutions of tested peptides in RPMI-1640. For the positive control (100% viable cells), RPMI-1640 medium was used instead of the peptide solution, whereas for the negative control (0% viable cells) medium replaced both peptide and cells. Test plates were incubated at 37°C and 5% CO_2_ overnight, after which MTT metabolic marker in PBS (5 mg/ml) was introduced into each well. After an additional 4 h incubation, the reaction was stopped by adding isopropanol supplemented with 0.04 M HCl, in which the formazan crystals (formed as a result of MTT reduction in presence of metabolically active cells) were dissolved, and OD at 540 nm was measured. Background absorbance at 690 nm was subtracted from the results to countervail any unspecific OD fluctuations. The % of surviving cells was found as:

(2) $$Survival(\%)=\frac{\left(OD_{sample}-OD_{0\%\;viable}\right)}{\left(OD_{100\%\;viable}-OD_{0\%\;viable}\right)}\times 100\%,$$

where OD_sample_, OD_0% viable_, and OD_100% viable_ are respectively values of the OD at 540 nm minus the OD at 690 nm for the test sample and negative or positive controls. IC_50_ toxicity values were determined by nonlinear regression analysis of the dose response curves using Sigma Plot 11 (Systat Software Inc., USA). The experiments were repeated three times, with 3 parallels of samples and 4–6 parallels of controls each time.

#### Trypan Blue Dye Exclusion Assay

The cytoplasmic membrane of living cells is impermeable to the trypan blue dye; hence the reason trypan blue staining is a common method to assess cell viability (Strober, 2015). Living cells exclude the dye, so that their cytoplasm remains clear. When damaged cells take up the dye, it binds to intracellular proteins and renders them a blue color. Cells suspended to a concentration of 1 × 10^6^ per ml in RPMI-1640 medium (serum-free) were incubated with various concentrations of the peptides of interest for various periods of time at 37°C and 5% CO_2_. Then they were stained with 0.2% trypan blue dye solution in PBS for 3 min at room temperature, and dead and living cells were counted using a hemocytometer. Percentage of viable cells was calculated as follows:

(3) $$Viable\;cells\;(\%)\;=\frac{total\;number\;of\;viable\;cells}{total\;number\;of\;cells}\times 100\%,$$

Experiments were repeated three times before plotting time-killing curves. For each time point, data are presented as mean with standard deviation (SD).

#### Apoptosis Detection Assay

Annexin V-Cy3 Apoptosis Detection kit (Sigma, USA) was used to distinguish between cells dying *via* necrosis and cells entering apoptosis. This kit includes a combination of red and green fluorescent dyes; 6-Carboxyfluorescein diacetate (6-CFDA) is hydrolyzed by esterases inside viable (still functional) cells, producing the green fluorescent compound 6-carboxyfluorescein. Annexin-Cy3.18, giving red fluorescence, binds to phosphatidylserine which only becomes exposed in the outer leaflet of the plasma membrane for cells entering the apoptotic process, or if the plasma membrane is damaged during necrosis. Thus, living cells are only stained green, necrotic ones are only stained red, while cells in the early stages of apoptosis are stained by both dyes.

To assess the effects of the peptides, cells were incubated in their presence for 2 h under the same conditions as described for trypan blue assay. Cells were washed twice with PBS and then resuspended to the initial volume in double label staining solution prepared according to the manufacturer’s instruction (1 μg/ml of Annexin-Cy3.18 and 0.5 mM of 6-CFDA in 1 × binding buffer). After 10 min incubation at room temperature cells were washed from excess dyes with 1 × binding buffer and analyzed under the luminescent microscope (Leica DM2500, Leica Microsystems, Germany). Several fields of vision were observed for each sample, ensuring that the total number of sorted cells was not less than 400. The resulting percentage of viable, apoptotic, and necrotic cells are shown as mean with SD, calculated based on three independent experiments.

#### Antitumor Activity Examination *In Vivo*

To assess the antitumor potential of peptides *in vivo* their effect on survival of mice inoculated with Ehrlich ascites carcinoma (EAC) cells was investigated. F1 hybrid (C57BL/6J x CBA/J) 5 months old male mice with body weight of 28 ± 2 g were used for the testing. Mice were housed in one experimental group (eight animals) per cage and were maintained at constant room temperature and 12:12-h light–dark cycle with *ad libitum* access to water and food, in accordance with laboratory animal welfare standards. EAC cells were kindly supplied by Dr. E.P. Kiseleva (Immunology Department of the Institute of Experimental Medicine, Saint Petersburg, Russia) and were maintained by transplanting ~10^7^ cells intraperitoneally from a mouse with ascites to a healthy mouse every 10–14 days. For the experiment 0.5–1.0 ml of ascitic fluid containing EAC cells were taken from the abdominal cavity of the mouse on the 10^th^ day after intraperitoneal tumor inoculation. Cells were washed twice with sterile PBS (by centrifuging at 300 g at 4°C), then counted using a hemocytometer and diluted down to 1 × 10^6^ cells per ml. On Day 0 experimental mice were subcutaneously injected with 0.2 ml of the prepared EAC cells suspension in PBS (2 × 10^5^ cells per mouse) in the region of their upper back and randomly divided into groups of eight animals. Experimental groups had been receiving various doses of peptide dissolved in deionized water; the control group had been treated with deionized water alone. The recombinant ChBac3.4-1-COOH has been used in these experiments in doses of 1 or 100 μg per animal. The peptide was administered intraperitoneally in a volume of 0.1 ml per mouse; injections were made once per week for 1 month starting from Day 1. Survival time in experimental groups was compared with control *via* Mann–Whitney U-test (p < 0.05). Based on survivability data Kaplan–Meier curves were plotted and log rank test was performed.

### Ethics Statement

In this study erythrocytes and mononuclear cells used for evaluation of cytotoxic activity of the peptides, were obtained from a peripheral blood of healthy donors. Blood collection was performed in accordance with the protocol 1/20 from 27.02.2020 approved by the Ethical Committee of the Institute of Experimental Medicine. Written informed consent was obtained from all volunteers. The animal study was reviewed and approved by the same Ethical Committee.

## Results

### Effects Against Bacteria

#### Antimicrobial Activity Against Planktonic Bacteria

Minimal inhibitory concentrations (MICs) of the designed variants of ChBac3.4 were measured against a number of Gram-positive and Gram-negative bacteria including drug-resistant clinically isolated strains using broth microdilution assay (**Table 2**). Unusually for PR-AMPs, the native ChBac3.4-NH_2_ and most of its analogues has an appreciable activity against the former. Modifications that maintained a comparable length to the native peptide did not result in a significant difference in activity. Two variants which showed a slight improvement in the geometric mean of MICs (GMIC), used as the general assessment value (Orlov et al., 2019), were ChBac3.4(Н-), with the histidine deletion, and RFR-ChBac3.4(1-14)-NH_2_, the truncated analogue with an additional *N*-terminal RFR triplet, albeit no more than a 2-fold reduction was sometimes observed. Comparing the effect of *C*-terminal amidation, the fluctuations of individual MICs between amidated and non-amidated variants were generally minor, so there is no evidence suggesting that this particular feature plays a relevant role in the antimicrobial action, considering either the ChBac3.4 or ChBac3.4-1 series. Furthermore, while the GMIC of ChBac3.4-NH_2_ was lower than that of ChBac3.4-COOH, the GMIC of ChBac3.4-1-NH_2_ was on the contrary higher than that of ChBac3.4-1-COOH.

**Table 2:** Antimicrobial activity of СhBac3.4 variants against Gram-positive and Gram-negative bacteria.

	MIC^a^ (μM) against bacteria								
	Gram-negative:					Gram-positive:			GMIC^b^ *total*[Gr-|Gr+]
Peptides	*E. coli* ML-35p	*A. baumannii* 7226/16	*K. pneumoniae* ESBL 344/17	*P. aeruginosa* MDR 522/17	*E. coli* ESBL 521/17	*S. aureus *SG-511	*MRSA*ATCC 33591	*S. aureus* 1399/17	
ChBac3.4-NH_2_	2	4	4	16	4	0.5	16-32	8	***4.6*** [4.6|4.6]
ChBac3.4-COOH	2	4-8	4-8	32	4	0.5	32	8-16	***6.0*** [6.2|5.8]
ChBac3.4-1-NH_2_	4	4	8	32	8	0.25	16	8	***5.7*** [8.0|3.2]
ChBac3.4-1-COOH	2-4	4	8	16	4-8	0.25	16	8	***4.8*** [6.2|3.2]
RFR-ChBac3.4-1-NH_2_	4	8	4-8	16	8	0.5	8	4-8	***5.3*** [7.6|2.9]
ChBac3.4-2-COOH	4	8-16	8	16	4	0.5	8	8	***5.5*** [7.6|3.2]
ChBac3.4 (Н-)	4	8	2	8-16	2	0.5	16	4	3.9* [4.3|3.2]
ChBac3.4 (12-26)	64	32	64	128	32	8	64-128	32	***43.7*** [55.7|29.1]
ChBac3.4 (1-19)-NH_2_	4	16	8	64	4	4	64	32	***13.5*** [10.6|20.2]
ChBac3.4 (1-14)-NH_2_	8	16	16	64	16	2	16	128	***17.4*** [18.4|16.0]
RFR-ChBac3.4 (1-14)-NH_2_	1	8	8	16	2	0.5	8	4	3.7* [4.6|2.5]

a Minimal inhibitory concentrations (MIC) are shown as medians of 3–6 independent experiments carried out in triplicate.

b The geometric mean of MICs measured against different bacterial strains (GMIC) is used for an overall assessment of antimicrobial activity of tested ChBac3.4 variants. GMIC values lower than that of the native ChBac3.4 are marked with asterisks. GMICs against all tested bacteria are given in bold italic; GMIC values against Gram-negative (Gr-) and Gram-positive (Gr+) strains calculated separately are given in square brackets on the left and right, respectively.

The dynamics of antimicrobial action amongst truncated variants of ChBac3.4 was somewhat more representative. The *C*-terminal fragment of the molecule was as expected the least active of all tested peptides, in agreement with the data previously reported for the bovine bactenecines Bac5 and Bac7 (Skerlavaj et al., 1999; Tokunaga et al., 2001; Gennaro et al., 2002). The gradual shortening of the *N*-terminal part of ChBac3.4 revealed that the antimicrobial activity against Gram-positive bacteria is most sensitive to this type of deletion. ChBac3.4(12-26) lost activity across the spectrum, whereas shortening from the *C*-terminus in the ChBac3.4(1-19) fragment resulted in a drop of activity in particular against all three tested *S. aureus* strains, meanwhile in case of Gram-negative bacteria a drop was observed mainly against isolates of *P. aeruginosa* and *A. baumannii*. Interestingly, these were found to be the most active biofilm formers amongst the tested microbes. Considering the significantly broader activity spectrum that distinguished ChBac3.4 from the majority of other bactenecins described to date, it is possible that clues to this particular feature may be found in the downstream part of ChBac3.4 from residues 12–18.

The more dramatically shortened ChBac3.4(1-14) fragment loses activity with respect to the native peptide, against both Gram-positive and Gram-negative bacteria, even though it is generally not completely abrogated. However, the addition of an extra RFR triplet to the *N*-terminus of this truncated ChBac3.4 variant effectively restored its antimicrobial potency to a level comparable to the full-length peptide: the overall GMIC was actually slightly lower than that of the native ChBac3.4. Such an increase in activity is in agreement with literature data on SAR for bovine Bac5 (Gennaro et al., 2002). In this case, however, it is hard to tell if it is the presence of an additional RFR motif that contributes the most to restoration of activity, or simply the increase in charge from +6 to +8. It has often been reported in SAR studies of cationic AMPs in general, that the net charge has a strong positive correlation with the antimicrobial potency, generally above a threshold of 4/5+ and up to a maximum threshold (Takahashi et al., 2010). On one hand, fact that the (1-19) fragment has the same charge as the (1-14) fragment, but a higher activity argues against a mere charge effect; on the other, addition of RFR on the *N*-terminus of a full-length ChBac3.4-1 analogue, already having a net charge of +8, does not lead to a notable increase in its antimicrobial potency. Interestingly, whereas the overall activity level of the RFR-ChBac3.4-1-NH_2_, represented by its GMIC, was not apparently improved with respect to the native ChBac3.4, it was somewhat better against *S. aureus* strains and somewhat worse against *E. coli*. In fact, when GMICs against Gram-negative and Gram-positive strains are considered separately (see **Table 2**), modifications possessing the additional RFR triplet [e.g. RFR-ChBac3.4-1-NH_2_ and the shortened RFR-ChBac3.4(1-14)-NH_2_] clearly show the most prominent improvement of activity against Gram-positive bacteria amongst all tested variants. The phenomenon of diminished antimicrobial effect against some of the resistant Gram-negative strains accompanied by the increased action against Gram-positive ones was observed also for the ChBac3.4-2-COOH modification. When two tryptophan residues were introduced into the native ChBac3.4 sequence as in the sequence of some tryptophan-rich peptides, such as indolicidin or tritrpticin, a “flattening” of activity across the spectrum was observed that could be an indication of decreased selectivity.

#### Combined Antimicrobial Action With Antibiotics

It was found (see paragraph *Cytotoxic Action* below) that addition of an extra RFR motif to the ChBac3.4(1-14)-NH_2_ quite peculiarly restored only its antibacterial properties, but not its toxicity for eukaryotic cells, so that the RFR-ChBac3.4(1-14)-NH_2_ peptide was considered the most promising antimicrobial on the basis of the combination of its individual activity and selectivity toward bacteria. As reported previously (Zharkova et al., 2019) the native ChBac3.4 has a synergic antimicrobial action with clinically used antibiotics, which extends to bacteria demonstrating a moderate to high level of resistance to the latter. It was therefore of interest to verify whether the truncated RFR-ChBac3.4(1-14)-NH_2_ modification retained the capacity to enhance the effectiveness of classic antibiotics.

The combined effect of RFR-ChBac3.4(1-14)-NH_2_ with the antibiotics oxacillin, meropenem, erythromycin, amikacin and ofloxacin was tested against five drug-resistant isolates: *E. coli* ESBL 521/17, *A. baumannii* 7226/16, *P. aeruginosa* MDR 522/17, *K. pneumoniae* ESBL 344/17 and *S. aureus* 1399/17. Corresponding minimal FICIs are given in **Table 3**, accompanied by FICIs previously evaluated for native ChBac3.4 in a recent article (Zharkova et al., 2019) presented for easier comparison. All cases of synergistic interaction found for the original ChBac3.4 were confirmed for the shortened RFR-ChBac3.4(1-14)-NH_2_ analogue, with the exception of the combination with meropenem against *S. aureus*. On the other hand, RFR-ChBac3.4(1-14)-NH_2_ compensates by demonstrating four cases of synergy against Gram-negative bacteria where ChBac3.4-NH_2_ showed only additivity: with erythromycin against *A. baumannii*, with meropenem against *P. aeruginosa* and with ofloxacin against *K. pneumoniae* and *P. aeruginosa*. Furthermore, in the majority of synergy cases concerning Gram-negative bacteria, the minimal FICI values for combinations with RFR-ChBac3.4(1-14)-NH_2_ were lower than the corresponding FICIs for combinations with native ChBac3.4, This suggests that the amplification effect for use in tandem with antibiotics emerges more prominently for RFR-ChBac3.4(1-14)-NH_2_. Only *S. aureus* 1399/17 shows an opposite trend, even if it is not so marked. This may be seen as an additional argument supporting the role of the *C*-terminal region of ChBac3.4 for its action on Gram-positive bacteria.

**Table 3:** Antimicrobial activity of the RFR-ChBac3.4(1-14)-NH_2_ analogue in combination with conventional antibiotics against drug-resistant clinical isolates compared to native ChBac3.4.

	Minimal FICIs^a^ of antibiotic(AB)\AMP combinations against drug-resistant clinical isolates									
	Gram-negative:								Gram-positive:	
	*E. coli* ESBL 521/17		*A. baumannii* 7226/16		*P. aeruginosa* MDR 522/17		*K. pneumoniae* ESBL 344/17		*S. aureus* 1399/17	
AB\AMP	ChBac3.4^b^	RFR-ChBac3.4(1–14)	ChBac3.4^b^	RFR-ChBac3.4(1–14)	ChBac3.4^b^	RFR-ChBac3.4(1–14)	ChBac3.4^b^	RFR-ChBac3.4(1–14)	ChBac3.4^b^	RFR-ChBac3.4(1–14)
OX	1.12	1.12	1.12	1.12	1.12	1.12	1.12	1.12	**0.25**	**0.31^–^**
MEM	1.0	1.12	**0.5**	**0.5^=^**	0.62	**0.38^!^**	1.0	0.75	**0.31**	0.62**^!^**
ERY	**0.38**	**0.38^=^**	0.75	**0.25^!^**	**0.38**	**0.19^+^**	**0.25**	**0.06^+^**	**0.5**	**0.28^+^**
AMK	0.62	0.75	**0.5**	**0.19^+^**	**0.5**	**0.38^+^**	**0.38**	**0.31^+^**	**0.125**	**0.19^–^**
OFL	0.75	0.56	**0.5**	**0.5^=^**	0.75	**0.31^!^**	0.75	**0.25^!^**	**0.125**	**0.5^–^**

AMP, antimicrobial peptide; AB, antibiotic; OX, oxacillin; MEM, meropenem; ERY, erythromycin; AMK, amikacin; OFL, ofloxacin.

a Fractional Inhibitory Concentration Indices (FICI) values are medians of 3–4 independent experiments; minFICI > 2 indicates antagonism, 1 < minFICI ≤ 2 shows independent action, 0.5 < minFICI ≤ 1 corresponds to additivity, minFICI ≤ 0.5 denotes synergy; synergy cases are set off in bold type.

b Data on ChBac3.4 antimicrobial activity in combinations with antibiotics, taken from (Zharkova et al., 2019), are sown for easier comparison.

Lower FICIs illustrate more intense synergistic interaction. The cases where synergy demonstrated by an antibiotic in combination with RFR-ChBac3.4(1-14)-NH_2_ is similar to, or stronger or lower than that in combination with native ChBac3.4 are marked “=“, “+” and “–”, respectively. Cases of synergy that exclusively emerged (or were lost) for combinations with RFR-ChBac3.4(1-14)-NH_2_ are indicated by “!”.

#### Effects on Permeability of Bacterial Membranes

**Figure 2:**
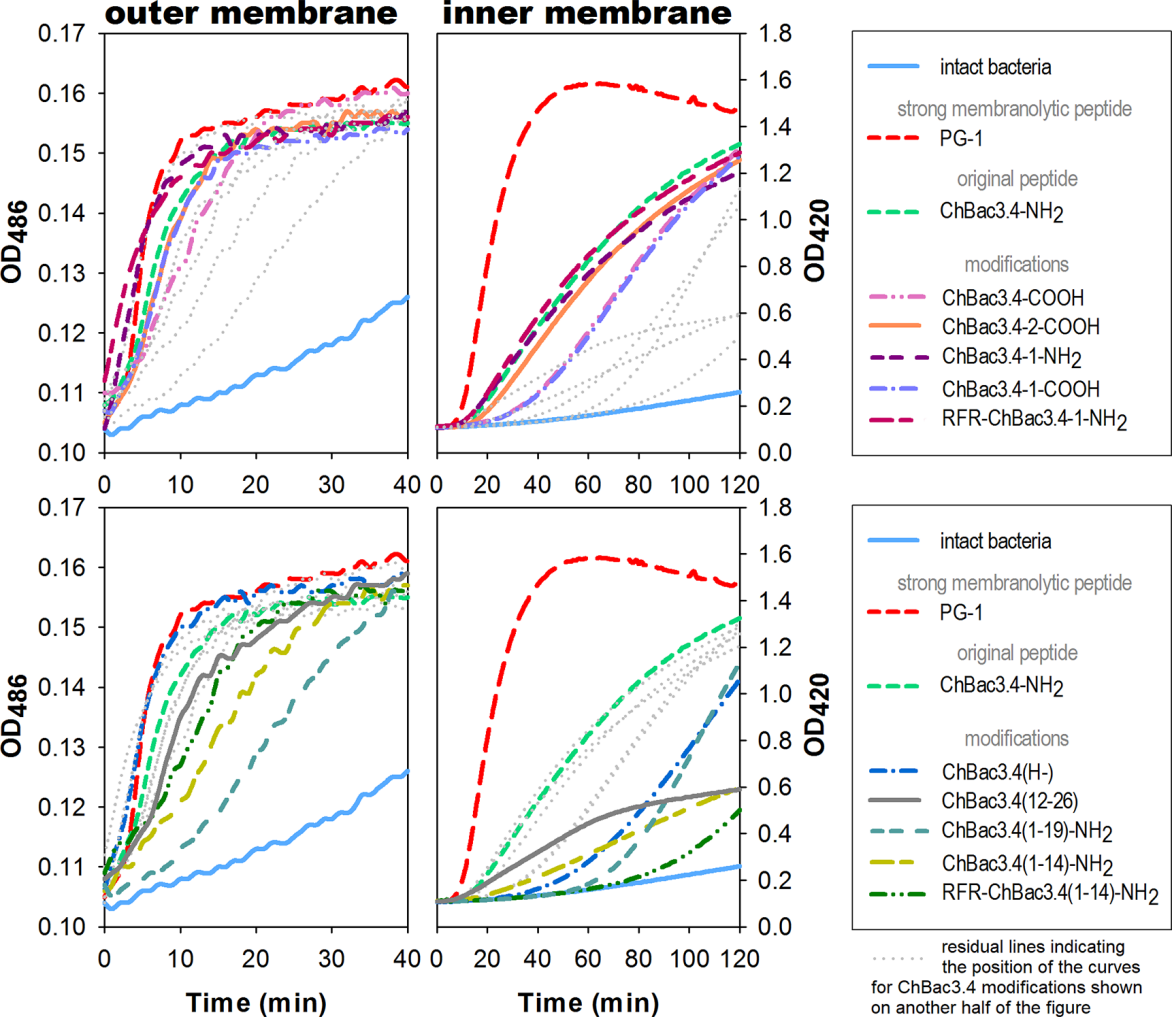
Effects of ChBac3.4 modifications on membrane permeability of *E.coli* ML-35p. The left panel shows changes in permeability of the outer membrane for the chromogenic marker nitrocefin; the right panel demonstrates changes in permeability of the inner membrane for the chromogenic probe molecule *o*-nitrophenyl-β-*D*-galactoside (ONPG). Concentrations of AMPs equal to 2×MIC were used to assess their membranolytic potential. Curves illustrate the accumulation of the colored products after hydrolysis by corresponding bacterial enzymes (periplasmic β-galactosidase for nitrocefin or cytoplasmic β-galactosidase for ONPG), that can only occur when membranes damaged by tested peptides allow access of the substrate to the enzyme. The lag time required for initiating the rise in optical density (OD), the rising period up to a plateau, and the slope of the curves during this period all characterize the velocity and the extent of the damaging.

Spectrophotometric monitoring of membrane permeability for chromogenic markers revealed that all tested variants of ChBac3.4 start to inflict significant damage to the outer membrane of *E. coli* ML-35p within the first 10 min from initial contact (**Figure 2**, left panel). Most act almost as effectively as the highly membranolytic porcine AMP PG-1, used as a reference for prominent membrane damaging. For all peptides, nitrocefin was fully degraded by bacterial periplasmic β-lactamase within 40 min, indicating an extensive effect on the bacterial outer membrane barrier. While it is not surprising that the less charged ChBac3.4(1-14) and (1-19) are less effective in permeabilizing this barrier, the poorly charged, truncated *C*-terminal fragment ChBac3.4(12-26) is quite effective, supporting its role in mediating activity against Gram-positive bacteria. The impact of the peptides on the integrity of the inner membrane is however not as uniform (**Figure 2**, right panel). ChBac3.4-2-COOH, ChBac3.4-1-NH_2_, and RFR-ChBac3.4-NH_2_ were as active as the native ChBac3.4-NH_2_. The tilt angles of the curves illustrating the effect of the least antimicrobially active ChBac3.4 fragments—ChBac3.4(12-26) and ChBac3.4(1-14)-NH_2_—were predictably the smallest, and their curves never raised close to the plateau level within 2 h of monitoring. This is not just an effect of lower charge, as the RFR-ChBac3.4(1-14)-NH_2_ was the least effective in permeabilizing inner membrane, while the similarly charged ChBac3.4(1-19)-NH_2_ had a steep, if delayed, tilt angle. What is apparent, for some of the modifications which exerted prominent antibacterial action in broth microdilution test, is that membrane permeabilization was noticeably postponed compared to the original ChBac3.4-NH_2_, albeit progressing quickly once it starts. In some cases [e.g. ChBac3.4(1-19)-NH_2_, 40 min delay] this might be ascribed to slow outer membrane permeabilization, but not for others [e.g. ChBac3.4(H-), 20 min delay, RFR-ChBac3.4(1-14)-NH_2_, 60 min delay]. We noticed a 15–20 min delay also for non-amidated variants of ChBac3.4 and ChBac3.4-1. As indicated above, it seems improbable, that this is just the effect of the decreased net charge upon the initial stage of peptide adsorption on the bacterial membrane due to accordingly diminished electrostatic attraction. Moreover, it apparently was not the case regarding the permeability of outer membrane. One may speculate that some other stages of AMP/membrane interaction may be altered, such as *i)* the transition through the peptidoglycan shell, *ii)* aggregation of peptide molecules on the membrane surface, *iii)* their reorientation and insertion within the bilayer. We cannot exclude that for longer delays membrane damage may be a collateral effect of the peptide killing the bacterium by inactivating an intracellular target.

#### Inhibition of Biofilm Formation

As the addition of an RFR triplet to the *N*-terminus of the (1-14) ChBac3.4 fragment induced the most prominent positive effect on antimicrobial activity amongst the tested modifications, we further analyzed the capacity of RFR-containing ChBac3.4 variants to inhibit biofilm formation. As already mentioned, amongst the tested bacteria the most prominent at forming biofilms were *P. aeruginosa* MDR 522/17 and *A. baumannii* 7226/16; hence the potential to prevent biofilm formation was investigated against these two strains (**Table 4**). The crystal violet assay was used to quantitatively assess biofilms formed in presence of different concentrations of the peptides. As well as the native ChBac3.4-NH_2_, RFR-ChBac3.4-1-NH_2_ also fully prevented biofilm formation at concentrations equal to 1–2×MIC. The antibiofilm activity of the shortened RFR-ChBac3.4(1-14)-NH_2_ was slightly diminished, so that a concentration of 4×MIC was required for 100% biofilm inhibition.

**Table 4:** Effects of СhBac3.4 variants containing additional RFR-motif on biofilm formation.

	Minimal concentrations of peptides required for exerting the specified effectagainst the specified bacteria					
	ChBac3.4-NH_2_		RFR-ChBac3.4-1-NH_2_		RFR-ChBac3.4(1-14)-NH_2_	
Effect	*A. baumannii* 7226/16	*P. aeruginosa* MDR 522/17	*A. baumannii* 7226/16	*P. aeruginosa* MDR 522/17	*A. baumannii* 7226/16	*P. aeruginosa* MDR 522/17
100% inhibition of biofilm formation^a^	2×MIC	MIC	MIC	2×MIC	4×MIC	4×MIC
>50% decrease in the thickness of the formed biofilm	½ MIC	^1^∕_64_ MIC	¼ MIC	½ MIC	2×MIC	MIC
Statistically significant decrease in thickness of the formed biofilm^b^	^1^∕_16_ MIC	^1^∕_128_ MIC	¼ MIC	^1^∕_1024_ MIC	2×MIC	^1^∕_512_ MIC

MIC, minimal inhibitory concentration (see **Table 2**).

a No difference found by Mann–Whitney U-test (p < 0.05) between the sample containing AMP and the negative control of biofilm formation (growth medium without bacteria).

b A difference found by Mann–Whitney U-test (p < 0.05) between the sample containing AMP and the positive control of biofilm formation (bacteria without antimicrobial compounds in growth medium).

At lower concentrations, the action of the tested peptides resulted in a partial inhibition of the biofilm growth, with *A. baumannii* biofilm being the more resistant. RFR-ChBac3.4(1-14)-NH_2_ induced a statistically significant decrease in the thickness of the forming *A. baumannii* biofilm at no less than 2×MIC, where the biofilm was 53 ± 3% weaker than that in the intact control (Mann–Whitney U-test; p < 0.05). Native ChBac3.4 was capable of attenuating biofilm growth in *A. baumannii* at sub-MIC concentrations down to ^1^∕_16_ MIC, but showed over 50% effectiveness (56 ± 12% inhibition) only at ½ MIC. RFR-ChBac3.4-1-NH_2_ was effective down to ¼ MIC (59 ± 11% inhibition increasing to 88 ± 5% inhibition at ½ MIC).

*P. aeruginosa* biofilm turned out to be more susceptible to the effects of sub-MIC concentrations of ChBac3.4 and its variants. The native peptide itself exerted a statistically significant antibiofilm action at concentrations down to ^1^∕_128_ MIC, and the lowest concentration where the decrease in biofilm thickness was >50% was ^1^∕_64_ MIC (63 ± 9% inhibition). RFR-ChBac3.4-1-NH_2_ and RFR-ChBac3.4(1-14)-NH_2_ were effective in measurably decreasing biofilm formation at even lower concentrations [down to ^1^∕_1024_ MIC for RFR-ChBac3.4-1-NH_2_ and ^1^∕_512_ for RFR-ChBac3.4(1-14)-NH_2_], but had not reached 50% inhibition at ¼. The effect at ½ MIC was respectively 62 ± 1.5% inhibition for RFR-ChBac3.4-1 and 79 ± 5% inhibition for RFR-ChBac3.4(1-14)-NH_2_.

### Effects on Eukaryotic Cells

#### Hemolytic Activity

**Figure 3:**
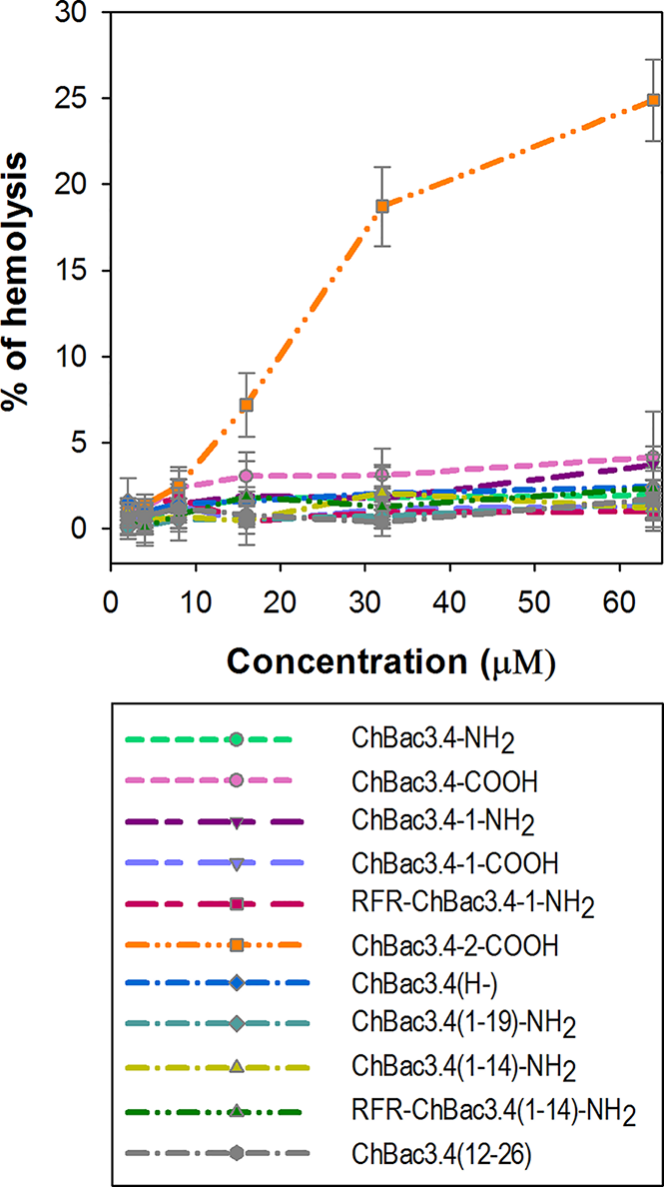
Hemolytic action of ChBac3.4 variants toward human red blood cells. Dots with error bars represent mean values and standard deviations derived from three independent experiments performed in triplicate.

The majority of AMPs are membranolytic, and even though eukaryotic cell membranes are less anionic than those of bacterial cells, have a lower membrane potential and are protected by the presence of cholesterol (Matsuzaki, 2009), they are considered to be a secondary target, affecting the selectivity of AMPs. In this respect, ChBac3.4 is more membrane active than other PR-AMPs, suggesting that this becomes a relevant issue. The hemolysis assay is widely utilized for the express analysis of the toxic properties of AMPs, relating to their membranolytic activity. It was previously shown that native ChBac3.4, similarly to other goat-derived bactenecins, is not hemolytic up to concentration 1–2 orders of magnitude higher than its MIC values against bacteria (Shamova et al., 2009; Shamova et al., 2016). We extended this analysis to the designed variants, and amongst tested ChBac3.4 modifications, only ChBac3.4-2-COOH demonstrated a clear tendency for dose-dependent lysis of human red blood cells (RBC), starting at ~ 7 ± 2% lysed erythrocytes at 16 μM and progressing up to 19 ± 2% at 32 μM and 25 ± 2% at 64 μM (**Figure 3**). The increased hemolycity of this peptide, even though it is moderate at concentrations tenfold the GMIC, may be due to its increased hydrophobicity due to the introduction of two tryptophan residues. For other ChBac3.4 variants there is no hint of an increased hemolytic activity up to 64 μM, as the < 5% hemolysis of RBC corresponds to the baseline (**Figure 3**).

#### Cytotoxic Action

The cytotoxic properties of ChBac3.4 variants were also characterized by using the MTT test against human peripheral blood mononuclear cells (PBMC) obtained from healthy donors, and two tumor cell lines: human erythroleukemia K562 and human histiocytic lymphoma U937 (**Table 5**). Unlike the situation with antimicrobial activity and hemolysis, the full-length ChBac3.4 variants showed decidedly more distinguishable shifts in their toxicity compared with the “parent” peptide, suggesting that changes in the primary structure influenced their interactions with eukaryotic cells more than with bacterial ones. ChBac3.4-1-NH2 was found to be the most cytotoxic amongst all tested analogues, considering both the tumor and nontumor cells. Its half maximal inhibitory concentration (IC_50_) was almost two times lower than that of native ChBac3.4-NH_2_ for each cell type investigated. RFR-ChBac3.4-1-NH_2_ and ChBac3.4-2-COOH variants showed a toxicity level comparable with that of ChBac3.4-1-NH_2_ against U937 cells. However, ChBac3.4-2-COOH activity toward K562 cells was only slightly enhanced compared with the original ChBac3.4-NH_2_, and neither PBMC, nor K562 cells were affected more by RFR-ChBac3.4-1-NH_2_ than by the latter. ChBac3.4-COOH and ChBac3.4-1-COOH were found to be less cytotoxic than their amidated counterparts against all tested cell types, which agrees with the observation that increasing cationicity over some point may start to affect toxicity toward eukaryotic cells rather than contributing just to antimicrobial effectiveness (Dathe et al., 2001; Takahashi et al., 2010). The histidine deficient analogue had a significantly lower cytotoxic action compared with native ChBac3.4, with a 42%–86% higher IC_50_, depending on cell type, resulting in a twofold increase in selectivity. As for the activity of truncated versions of ChBac3.4, all of them demonstrated a significant decrease in toxicity, which is more prominent for *N*-terminal fragments. This clearly shows that while this region is crucial for antibacterial action, it is not for cytotoxicity to animal cells.

**Table 5:** Cytotoxic action of СhBac3.4 modifications toward normal or tumor eukaryotic cells and resulting selectivity indices (SI).

	IC_50_ ^a^ (μM) of cytotoxic action toward			SI_h/b_ ^b^	SI_n/t_ ^c^
	normal cells:	tumor cells:		$\frac{IC_{50}^{PBMC}}{GMIC}$ [ratio to ChBac3.4^d^]	$\frac{IC_{50}^{PBMC}}{\sqrt{IC_{50}^{K562}∙IC_{50}^{U937}}}$ [ratio to ChBac3.4^d^]
Peptides	human PBMC	K562	U937		
ChBac3.4-NH_2_	18.6 ± 0.8	15.5 ± 0.3	8.1 ± 0.4	4.0 [=]	1.7 [=]
ChBac3.4-COOH	36.2 ± 6.9	24.4 ± 4.0	12.9 ± 0.3	6.0 [1.5]	2.0 [1.2]
ChBac3.4-1-NH_2_	12.1 ± 0.8	8.4 ± 1.1	4.5 ± 1.6	2.1 [0.5]	2.0 [1.2]
ChBac3.4-1-COOH	33.7 ± 3.2	16.5 ± 1.3	9.8 ± 0.5	7.0 [1.7]	2.7 [1.6]*
RFR-ChBac3.4-1-NH_2_	18.3 ± 4.1	20.0 ± 3.4	3.2 ± 0.4	3.5 [0.9]	2.3 [1.4]
ChBac3.4-2-COOH	20.2 ± 4.5	13.7 ± 2.3	4.1 ± 0.3	3.7 [0.9]	2.7 [1.6]*
ChBac3.4 (H-)	34.7 ± 7.3	25.0 ± 3.5	11.5 ± 0.5	8.9 [2.2]	2.0 [1.2]
ChBac3.4 (12-26)	43.3 ± 7.7	39.4 ± 2.3	17.0 ± 1.1	1.0 [0.2]	1.7 [1.0]
ChBac3.4 (1-19)-NH_2_	>64	28.0 ± 6.5	>64	>4.7 [>1.2]	~1.5 [~0.9]
ChBac3.4 (1-14)-NH_2_	>64	33.6 ± 4.1	>64	>3.7 [>0.9]	~1.4 [~0.8]
RFR-ChBac3.4 (1-14)-NH_2_	>64	39.4 ± 6.6	>64	>17.3[>4.3]*	~1.3 [~0.8]

PBMC, peripheral blood mononuclear cells; K562, human erythroleukemia cells; U937, human histiocytic lymphoma cells.

a Half maximal inhibitory concentrations (IC_50_) of cytotoxic action were calculated using Sigma Plot Standard Curve Analysis based on data of three independent MTT-tests and are shown as mean ± standard deviation.

b Selectivity index illustrating how much the peptide “prefers” damaging bacterial cells over normal human cells (SI_h/b_). It was determined by dividing IC_50_ toward human PBMC by the geometric mean of minimal inhibitory concentrations measured against different bacterial strains (GMIC, see **Table 2**).

c Selectivity index demonstrating if toxic effects of the peptide toward tumor cells are higher than toward normal eukaryotic cells (SI_n/t_). It was calculated as per the indciated formula, considering the geometric mean of ICs_50_ toward both tested types of tumor cells.

^d^ To assess the improvement in either types of selectivity, the ratios of the corresponding SI of the peptide of interest to the corresponding SI of the native ChBac3.4-NH_2_ are shown in square brackets. The best improvement results are marked with asterisks.

We estimated the selectivity of ChBac3.4 variants comparing either their *in vitro* impact on bacterial cells with respect to human PBMC, or toward tumor cell lines with respect to PBMC, and calculated the appropriate selectivity indices (**SI_h/b_** and **SI_n/t_** see **Table 5**). These provide a sufficiently plausible assessment of the width of the ‘therapeutic window’ in case of their possible future application as either antibacterial or antitumor agents. To summarize the overall effects studied against different types of eukaryotic or bacterial cells we used the geometric means of measured parameters (IC_50_ or MIC). Notably, both modifications leading to improved antimicrobial potency, namely ChBac3.4(H-) and RFR-ChBac3.4(1-14)-NH_2_, were also found to be more selective toward bacteria than the original ChBac3.4-NH_2_; RFR-ChBac3.4(1-14)-NH_2_ had the best SI_h/b_ in this regard (almost 20), enhanced by a factor of over 4 with respect to the native peptide. Gains in nontumor/tumor cells selectivity were less sizable, with a most favorable result being 1.6 times increase in selectivity for ChBac3.4-2-COOH and ChBac3.4-1-COOH. Interestingly, both of these peptides are somewhat more hydrophobic than the native ChBac3.4 (based on increased elution times during reversed-phase chromatography). The dramatic role of hydrophobicity in cytotoxic potency of AMPs is well-known (Wieprecht et al., 1997; Takahashi et al., 2010; Teixeira et al., 2012), and probably in some cases, below a certain threshold, it may quite handily enhance toxicity toward tumor cells more than that toward normal cells.

**Figure 4:**
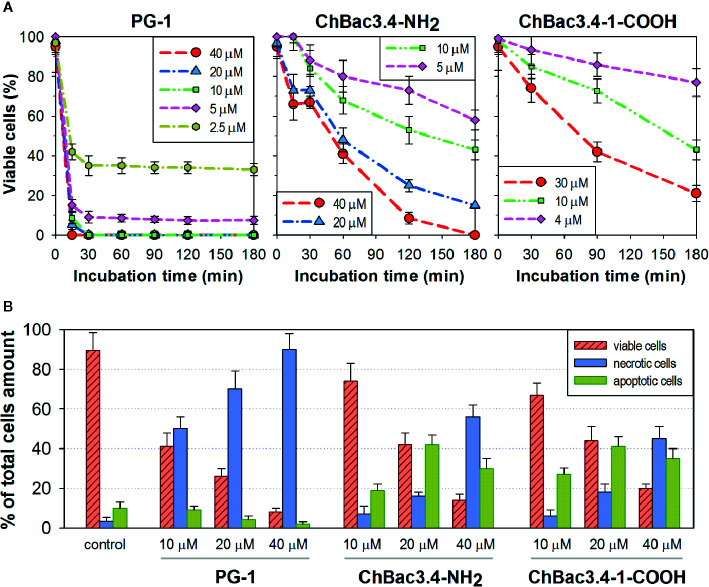
Dynamics of cytocidal action of ChBac3.4-1-COOH on K562 cells in comparison with the membranolytic porcine PG-1 and native ChBac3.4. **(A)** Time-killing curves illustrating the development of cytocidal effect for various concentrations of the peptides during the first 3 h of incubation, based on the data from the trypan blue dye exclusion assay. **(B)** Bar chart showing the percentage of cells dying *via* necrosis or apoptosis in the presence of different concentrations of the peptides.

Results on selectivity and individual effectiveness toward both bacterial and tumor cells indicate that ChBac3.4-1-COOH merits a further analysis of cytotoxic activity. The dynamics of cytocidal action during the early stages of the peptide’s contact with K562 cells was observed using the trypan blue dye exclusion assay in comparison with the native ChBac3.4, and with PG-1 used as an exemplary membranolytic AMP (**Figure 4A**). PG-1 showed a swift and pronounced effect, observable in the first 30 min of exposure; however at the later time points the relative percentage of stained and viable cells remains almost unchanged. Both native ChBac3.4-NH_2_ and modified ChBac3.4-1-COOH induced a much more gradual reduction of the viable cells population. Within 3 h of observation, the killing curve for ChBac3.4 reached its plateau only at the highest tested concentration, and the de-amidated ChBac3.4-1 variant progressed even more slowly. The ratio of apoptosis to necrosis amongst cells exposed to the peptides was quantitated using the Annexin V-Cy3 Apoptosis Detection kit (**Figure 4B**). The prominent, dose-dependent prevalence of necrosis over apoptosis demonstrated by PG-1 points to a clear positive correlation between necrosis and the membranolytic potency of this peptide. For ChBac3.4 and ChBac3.4-1 de-amidated version, necrosis starts to override apoptosis only at higher concentrations, indicating that the cytotoxic action of these peptides relies more closely on interactions with cellular targets (likely inside the cell) than on membrane disruption. ChBac3.4-1-COOH action results in a higher percentage of cells dying *via* apoptosis and lower percentage of cells dying *via* necrosis, which accounts for its higher apoptosis/necrosis ratio at high concentration compared with the native peptide, denoting a possible decrease in membranolytic potential and/or possible strengthening of intracellular activity.

#### Antitumor Activity *In Vivo*

The antitumor potency of de-amidated ChBac3.4-1-COOH was verified *in vivo*. Recombinant ChBac3.4-1-COOH was applied in these experiments. To certify that there is no differences between the peptide produced by a recombinant expression system and chemically synthesized one we have compared their antibacterial activity and cytotoxicity toward mammalian cells *in vitro*. The antimicrobial activity of the peptides was estimated by the broth microdilution assay against *A. baumannii* 7226/16, *P. aeruginosa* MDR 522/17, *K. pneumoniae* ESBL 344/17, *E. coli* ESBL 521/17, and the correspondent MICs for both peptides were not statistically different (see **Supplementary Table 2**). The results of MTT test performed to evaluate IC_50_ values of the peptides’ action on normal PMBC or K562 cells also demonsrated no differences between chemically synthesized and recombinant ChBac3.4-1-COOH (see **Supplementary Table 3**).

**Figure 5:**
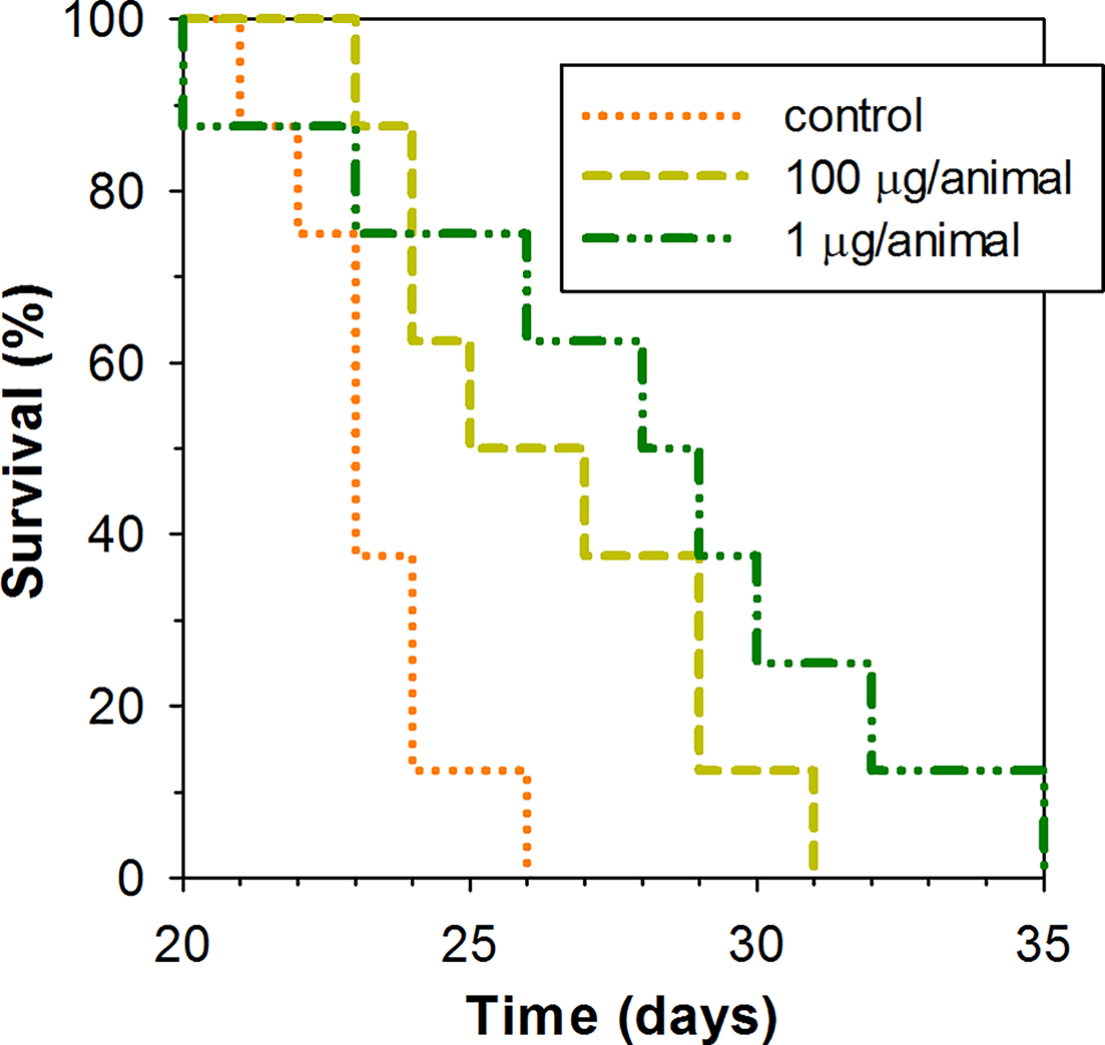
Kaplan–Meier survival curves of mice treated with ChBac3.4-1-COOH in a subcutaneous Ehrlich ascites carcinoma model. Mice in experimental groups were injected with specified doses of the peptide dissolved in 0.1 ml of deionized water once per week. The control group was injected with the same amount of deionized water. A log rank test shows statistically significant difference between both 100 μg receiving group (p = 0.039) and 1 μg reseiving group (p = 0.034) and control.

A low dose of 1 μg of peptide per animal and higher dose of 100 μg of the peptide per animal were examined for their capacity to lengthen the survival time of mice subcutaneously inoculated with EAC cells in their upper back. Peptide was administered intraperitoneally in the specified dosage once per week. As Kaplan–Meier survival curves demonstrate (**Figure 5**), ChBac3.4-1-COOH does indeed exert a moderate positive effect *in vivo* on the lifespan of animals with carcinoma, at both the low and high doses. The average survival time in a group receiving 100 μg of peptide per mouse was 26.5 ± 2.9 (mean ± SD) days, and in a group receiving 1 μg per mouse it was actually slightly longer, 27.9 ± 4.8 days, which in both cases was statistically greater than in the control group, having an average survival time of 23.2 ± 1.5 days (Mann–Whitney U-test; p < 0.05). Median survival times were 25, 28, and 23 days respectively. Survival time improvement was also confirmed by the log rank test; a group treated with 100 or 1 μg dose of ChBac3.4-1-COOH showed a difference with untreated control with p = 0.039 and 0.034, respectively. However, no indications of a strong correlation between ChBac3.4-1-COOH dosage and the magnitude of the effect were observed. As no visible signs of toxic action at the utilized doses for CHBac3.4-1-COOH were previously found in a standard acute toxicity test (data not shown), it seems unlikely that this situation could be explained by the peptide’s toxicity starting to override the benefits of its antitumor effect at the higher concentration. An explanation could be that the observed positive impact on mice survival time occurs rather due to some less concentration-dependant immunomodulation mechanisms, which may or may not involve a peptide interaction with the tumor cells, than due to a direct cytotoxic action of the peptide toward tumor cells themselves.

## Discussion

Resistance of bacteria to commonly used antibiotics is rapidly growing, and mortality from infections caused by antibiotic-resistant bacteria is projected to reach 10 million people per year by 2050. This threat defines the urgent need to discover new classes of antibiotics. During evolution, multicellular organisms have developed many tools to fight pathogenic bacteria. The innate and adaptive immune systems both provide anti-infective defense making use of a wide arsenal of antimicrobial molecules. The cationic antimicrobial peptides of the innate immune system quickly and effectively inactivate pathogens and have therefore been attracting the attention of researchers as prototypes for novel potent antibiotics for decades. Studies progressing since their first identification revealed that their structural diversity gives rise to the plethora of intricate mechanisms and biological functions, not limited to non-specific membrane disruption and direct bacterial killing (Schmitt et al., 2016; Kumar et al., 2018; Haney et al., 2019). Up to now, however, the detailed and extensive consideration placed on many of these molecules has been thwarted by toxicity with respect to human cells, which limits the practical use of the most effective AMPs. This has determined the creation of hundreds of structural variants in the hope of obtaining molecules with optimal features for medical applications. To this is added the high cost of production of peptides, in particular of those of the disulfide-bonded structural class, whereas the simpler linear structural class of AMPs suffers from instability in biological fluids, which poses another problem to the introduction of the peptides as anti-infective agents. In this respect, the smaller group of proline-rich AMPs (PR-AMPs) demonstrates an array of advantages: exceptionally low toxicity for mammalian cells, a relatively simpler procedure for chemical synthesis in good yields, and relatively good stability due to high content of proline, making these cationic peptides less sensitive to the cleavage by host or microbial proteases.

Several promising potential therapeutics have been developed on the basis of insect PR-AMPs—Api88, Api137, Onc72—that show a high protective capacity in *in vivo* models of lethal mouse infection caused by Gram-negative bacteria, including *E. coli* and a carbapenem-resistant *K. pneumoniae* (Knappe et al., 2012; Ostorhazi et al., 2014). The designer antibacterial dimeric peptide A3-APO is efficacious in different mouse models of multidrug-resistant wound and lung infections (Ostorhazi et al., 2011). Api88, Api137, and A3-APO demonstrate also a long-lasting post-antibiotic effect on Enterobacteriaceae and *Pseudomonas aeruginosa* (Holfeld et al., 2018). In addition, methods are being developed for their administration at effective doses (Schmidt et al., 2017). The peptides act not just as antimicrobials, but can also exert a potent immunomodulatory action, that can explain a higher efficacy sometimes observed for these peptides *in vivo* than would be expected from their antibacterial activity *in vitro* (Ostorhazi et al., 2011).

PR-AMPs of the innate immune system of mammals, in particular of cetartiodactyls, also represent a group of peptides with large prospects for practical use as novel tools to treat multiresistant infections. For thousands of years humans have used various food products obtained from domestic ruminants—milk from bovids of various types, including goats,—where such peptides can be present. In ‘80s an immunomodulatory, anti-inflammatory, neuroprotective, procognitive medication named colostrinin was created on the basis of a complex of proline-rich polypeptides isolated from ovine colostrum (Janusz et al., 1981). In the form of orally administered tablets, it was introduced into medical practice for the treatment of Alzheimer’s disease (Leszek et al., 1999).

There are data on an ability of the bovine hypothalamic proline-rich polypeptide galarmin to protect mice against lethal methicillin-resistant *Staphylococcus aureus* (MRSA) infection. On the face of it, this looks contradictory to the observation that PR-AMPs are predominantly active against Gram-negative bacteria *in vitro*. However, the protective effect of the peptide was in fact not due to a direct killing of bacteria, but *via* its immunomodulatory action: immunocompetent cell recruitment and modulation of the release of pro- and anti-inflammatory cytokines (IL-6, IL-8, IL-10, IL-1b, TNFa) (Durgaryan et al., 2012).

These data underline the importance of a comprehensive study of PR-AMPs of higher vertebrates for developing novel immunomodulatory and antimicrobial drugs. To date, the most well studied mammalian PR-AMPs are bovine bactenecins or their fragments, which exert a powerful action against a variety of multidrug-resistant Gram-negative pathogens (Mardirossian et al., 2019a; Price et al., 2019).

The present research is devoted to a comprehensive study of a biological activity of a caprine bactenecin ChBac3.4, which we have previously isolated from the goat leukocytes (Shamova et al., 2009) along with the other PR-AMPs—mini-ChBac7.5Nα and -β, ChBac5 (Shamova et al., 1999; Shamova et al., 2016)—and showed that they possess a marked activity against multidrug-resistant bacterial strains of Gram-negative hospital isolates (Zharkova et al., 2018). While minibactenecins and ChBac5 had similar capabilities with those of bovine ortologs, ChBac3.4 exerted a wider spectrum of antibacterial activity and therefore has been chosen for a more detailed study.

We have attempted to explore primary structural features of ChBac3.4 to identify those regions of the molecule responsible for the observed effects on bacterial or mammalian cells and possibly infer something about its molecular targets. By elaborating a set of rational ChBac3.4 structural variants we also hoped to find modifications with optimal characteristics for the future practical use as antibacterial and probably antitumor compounds.

The study of truncated variants of ChBac3.4 showed that a quite short *N*-terminal fragment of only 14 residues maintains some activity against Gram-negative bacteria. This observation is in accordance with the data reported by Mardirossian and colleagues for bovine Bac5 truncated analogues stating that the *N*-terminal fragments of Bac5 (1-25) and Bac5 (1-21) demonstrated a good antimicrobial activity as well as a potency to inhibit bacterial protein synthesis. Bac5 (1-17) was the shortest variant with appreciable antimicrobial activity and detectable effects on translation (Mardirossian et al., 2019b).

An addition of a second RFR triplet to the *N*-terminus of this truncated analogue of ChBac3.4 resulted in an increase in activity both against Gram-negative and Gram-positive bacteria. However, the permeabilizing effect of RFR-ChBac3.4(1-14)-NH_2_ on the *E. coli* ML-35p outer and inner membranes was less pronounced in comparison with the parent peptide, and even of the shorter ChBac3.4(1-14)-NH_2_ itself. For many AMPs altering the net charge leads to significant changes in their antimicrobial activity. A net charge of between +4 to +6 appears to be optimal, and a further increase in cationicity beyond the optimal range does not necessarily guarantee an improved antibacterial activity, while it can lead to the loss of selectivity for bacterial cells, as was shown with magainin 2 analogs (Takahashi et al., 2010). Here, despite the increased charge to +8 for RFR-ChBac3.4(1-14)-NH_2_, we believe that this is not the main reason for enhancement in its activity, but rather that a modulation of its interaction with other molecules plays an important role.

Previously described PR-AMPs caused an appreciable damage to bacterial membranes only at concentrations much higher than their MICs, so that their interplay with various other bacterial components was a necessary stage in dispatching their activity. As an initial step they interact with some bacterial membrane proteins: for related PR-AMPs such as bovine Bac5 and Bac7, as well as porcine PR39 this turned out to be during their translocation across the bacterial membrane by the inner membrane transporters SbmA and/or YjiL/MdtM of Gram-negative bacteria, without pore formation. As a next step these PR-AMPs bind to the ribosomal exit tunnel, inhibiting protein synthesis (Krizsan et al., 2014; Mardirossian et al., 2014; Krizsan et al., 2015; Roy et al., 2015), or alternatively to the DnaK bacterial chaperone, blocking protein folding (Scocchi et al., 2009; Zahn et al., 2013; Zahn et al., 2014).

As yet we have no direct evidence regarding the influence of the parent ChBac3.4 or its variants on bacterial protein synthesis, but given its homology to bovine PR-AMPs we are confident that they have a similar mode of action, involving entry of the truncated peptides into the ribosomal tunnel. The improved activity of RFR-ChBac(1-14)-NH_2_, however, is less likely to be due to an improved interaction with and translocation by the transporters required to translocate the peptide into the cytoplasm, as mentioned above, as the peptide is also effective against Gram-positive bacteria, which do not express the transporters. The importance of the *N*-terminal region for efficient transit through both the outer and cytoplasmic membranes of Gram-negative bacteria, using specific transport systems, has been determined for bovine Bac7 (Guida et al., 2015). We may speculate that interaction with the ribosomal tunnel was somewhat improved by virtue of adding RFR triplet to the *N*-terminus of the peptide, but this suggestion requires future investigations.

Despite the fact that differences in the antimicrobial activity of RFR-ChBac3.4(1-14)-NH_2_ compared to the parent ChBac3.4-NH_2_ were not remarkable, the combined action of the modified peptide with conventional antibiotics against drug-resistant *A. baumannii*, *P*. *aeruginosa, K. pneumoniae* ESBL was more powerful, with more cases of synergy and better FICI values, pointing to a better prospect for the application of this peptide in combination with common antibiotics.

We also observed potentially useful effects of the studied peptides on bacterial biofilm formation, one of the major bacterial strategies to evade unfriendly environmental factors, including the action of various antimicrobial compounds and of immune system of the host (de la Fuente-Núňez et al., 2016; Chen et al., 2018; Shahrour et al., 2019). Well over a half (65%–80% by different assessments) of clinical cases related with bacterial infections are associated with biofilms (Costerton et al., 1999; Jorge et al., 2012; Pletzer and Hancock, 2016). In this mode of growth bacterial cells are protected by a dense extracellular matrix, restricting their accessibility for immune effectors and clinically used antimicrobials alike. Moreover, persister cells existing within a biofilm are metabolically inactive, so they pose a serious challenge for conventional antibiotics (Jorge et al., 2012; Shahrour et al., 2019). For a little more than a decade, AMPs have been acknowledged as a promising source of antibiofilm agents as well as of antibacterials, although only a small fraction of identified AMPs have been adequately studied in this particular aspect to date (Dostert et al., 2019).

Unlike many conventional antibiotics, AMPs are capable of acting on metabolically inactive bacteria, since a main target of their action is bacterial membranes; this could be successfully exploited for the elimination of persister cells in combination with conventional drugs, which has been demonstrated by the synergistic interaction of the latter with AMPs against biofilms (Pletzer and Hancock, 2016; Chernysh et al., 2018). It was shown that various AMPs on their own not only eliminate planktonic bacteria, but also weaken the primary adhesion of bacterial cells to the surface, and within the established biofilms can impair the interaction of the elements of extracellular matrix and embedded bacterial cells. In addition, more specific mechanisms were revealed for a number of peptides demonstrating strong antibiofilm capabilities: they can modulate the motility of bacterial cells; affect the quorum sensing signaling; impede the work of the (p)ppGpp alarmone and, by extension, interfere with bacterial stringent response, and reduce the synthesis of various matrix elements or lead to their degradation (Pletzer and Hancock, 2016; Yasir et al., 2018; Casciaro et al., 2019; Shahrour et al., 2019).

In our research, we found that the native bactenecin ChBac3.4-NH_2_ has good antibiofilm potential. When the human catelicidin LL-37, which is well-known for its antibiofilm properties, was first identified in this capacity, its ability to prevent biofilm formation by *P. aeruginosa* was observed down to ^1^∕_128_ of its MIC, and reached about 50% inhibition starting from ^1^∕_16_ of MIC (Overhage et al., 2008). ChBac3.4-NH_2_ also shows antibiofilm activity against *P. aeruginosa* with the same range of sub-MIC concentrations and even demonstrated >50% inhibition down to ^1^∕_64_ of its MIC. Activity of ChBac3.4-NH_2_ and its full-length modification RFR-CBac3.4-1-NH_2_ against biofilm formed by *A. baumannii* isolate closely resembles that recently described for another member of the bactenicins family, bovine Bac7(1-35) (Dolzani et al., 2019), and manifests prominently down to ¼ of MIC. However, the impact of Bac7(1-35) on *P. aeruginosa* biofilm formation appears weaker than what we observed for ChBac3.4-NH_2_, as the significant and uniform inhibiting effect of Bac7(1-35) at concentration below ½ MIC was apparent only for one out of four tested *P. aeruginosa* isolates and this one isolate was also the most susceptible to Bac7(1-35) in the planktonic antibacterial assay (Runti et al., 2017). The decreased ability to fully prevent biofilm formation exhibited by RFR-ChBac3.4(1-14)-NH_2_ may indicate its lower performance against persisting cells due to impaired membranolytic properties. On the other hand, it is possible that the *C*-terminal part of ChBac3.4-NH_2_ may be relevant for full antibiofilm activity. Closer investigation of the exact mechanism of the antibiofilm action of ChBac3.4 is required to rationally design its modifications with improved activity in this particular aspect. This is also of interest from a broader perspective, as the majority of known antibiofilm peptides with identified specific targets significantly differ in their amino acid sequences from the proline-rich group of AMPs, so that these may harbor yet unknown mechanisms and targets. Identification of the corresponding structural motifs in PR-AMPs may subsequently be of use for developing chimeric variants that will incorporate the advantageous features of different classes of AMPs.

An analysis of the cytotoxic activity of the elaborated analogues of ChBac3.4 provided information on their selectivity for bacterial or tumor cells. The absence of *C*-terminal amidation led to a slight decrease in antibacterial activity as well as in cytotoxicity toward both normal and tumor cells. In this case, the differences might be partially explained by a higher net charge of the amidated analogues.

Although the most potent antibacterial variant, RFR-ChBac3.4(1-14)-NH_2_, showed only modest improvement of MIC values for planktonic bacteria and a lower efficacy against bacterial biofilms compared with the parent peptide, its negligible toxicity for human cells and the presence of the significant antimicrobial activity toward drug-resistant bacteria allows us to conclude that this peptide is quite promising in terms of practical application.

Examining the toxicity of the peptides toward tumor cells could furthermore reveal useful antitumor capacities of PR-AMPs. Cancer is another global concern, and despite recent advances in antitumor treatment, such as radiation therapy, targeted therapy or chemotherapeutic agents, is nowadays the second most common cause of death worldwide (Thundimadathil, 2012). Just as with infective diseases, there is a rising problem of resistance by cancer cells toward currently used anticancer drugs determining the vital necessity for developing novel anti-cancer drugs. Therapeutic agents with dual—antimicrobial and anti-tumor—action are of utmost interest since mortality of cancer patients is in many cases related with chronic infection, and vice versa patients with chronic infections and immunodeficiency are more susceptible to cancer (Felício et al., 2017; Otvos, 2017). AMPs are considered to have potential as therapeutics with such dual effect, as many AMPs have been also classified as anticancer peptides (ACPs). Among these are cecropins, magainin, melittin, buforin II, lactoferricin B, HNP-1-3, gomesin, tachyplesins, etc. (Papo and Shai, 2005; Balandin et al., 2016). If α-helical and β-sheet peptides have been studied as anti-cancer molecules, little is known about the anti-tumor activity of PR-AMPs. We had previously shown that ChBac3.4 exerted a distinct cytotoxicity toward some tumor cells including doxorubicin-resistant K562 cells *in vitro* (Shamova et al., 2009; Shamova et al., 2015). Here, the cytotoxic features of its structural analogues have been examined toward human erythromyeloid cell line K562, human hystiocytic lymphoma cells U937, human erythrocytes and PBMC. Most of the studied peptides exerted the cytotoxic activity against transformed cells without any appreciable hemolytic effects within the applied range of concentrations. The lack of the hemolytic activity is an important advantage of the investigated bactenecins over some ACPs [for instance BMAPs, melittin, etc. (Hoskin and Ramamoorthy, 2008)]. A variant showing decreased toxicity toward PBMC in comparison with the parent peptide, but a similar activity against tumor cells —ChBac3.4-1-COOH—was chosen for a more detailed study of its effects on mammalian cells using a membrane-active peptide PG-1 as a reference. Both ChBac3.4-NH_2_ and ChBac3.4-1-COOH had a delayed cytotoxic action compared to PG-1, which caused a rapid cellular death *via* necrosis, whereas the bactenecins at concentrations close to their IC_50_, initiated apoptosis in the target cells. In previous experiments we have observed an increase of a key effector in apoptosis—caspase-3 activity—in U937 and K562 cells treated with ChBac3.4 at a concentration close to IC_50_ (unpublished data). This suggests that both peptides rather affect intracellular processes than membrane integrity. In a murine model of Ehrlich carcinoma ChBac3.4-1-COOH prolonged the lifespan of the experimental animals at quite a low dose. Although the protective effect of the peptide was not remarkable, its action needs further investigation and may be improved. To receive a more powerful anticancer preparation several approaches might be applied: administration of the peptide in combination with a common anticancer drug [for example, as was shown for PG1 acting in synergy with doxorubicin against cancer cells *in vitro* (Zharkova et al., 2019)]; creation of chimeric peptides including tumor recognizing fragments for improving the selectivity index (SI); using nanocontainers for preventing proteolytic cleavage of the peptides and increasing their bioavailability for the target cells; computational redesign of novel bactenecin 3.4 analogues with improved activities by use of various computational tools, including those based on machine learning algorithms as described (Shoombuatong et al., 2018).

Taken together, our results confirm the feasibility and prospects of using ChBac3.4-derived peptides in medicine as anti-infective or antitumor drugs and indicate ways to promote the transition of these PR-AMPs from the bench to clinic.

## Conclusion

We have carried out a study of the antimicrobial and antitumor activity of a caprine proline-rich bactenecin ChBac3.4 (here indicated also as ChBac3.4-NH_2_) that we have previously isolated from the goat leukocytes, and investigated an array of its synthetic analogues. The parent peptide and some of its variants exert a potent antimicrobial activity against drug-resistant clinical isolates of Gram-negative bacteria from a group of ESKAPE pathogens, including meropenem-resistant and extended spectrum β-lactamase (ESBL) possessing strains; several ChBac3.4-NH_2_ analogues have a wider spectrum of activity expanded toward Gram-positive microorganisms as well. A short truncated variant (*N*-terminal fragment) ChBac3.4(1-14)-NH_2_ retains some antimicrobial activity, but completely lacks cytotoxicity toward mammalian cells, while a truncated *C*-terminal peptide, ChBac3.4(12-26)-NH_2_, in contrast, shows a significant decrease in antibacterial activity and to a lesser degree in its activity toward eukaryotic cells. Introduction of a second *N*-terminal triplet RFR onto ChBac3.4(1-14)-NH_2_ resulted in an enhancement of antibacterial efficacy without a rise in toxicity. RFR-ChBac3.4(1-14)-NH_2_ can be considered a promising candidate for a therapeutic development since it shows a distinct antimicrobial activity against planktonic bacteria and prevents biofilm formation by multidrug-resistant clinical isolates, along with a negligible toxicity toward human cells. The antimicrobial activity of RFR-ChBac3.4(1-14)-NH_2_ or other ChBac3.4-derived peptides can be significantly improved by applying them in combination with conventional antibiotics due to the prominent synergistic effects. The observed cytotoxic action of ChBac3.4 and its structural variants toward cultured tumor cells as well as *in vivo* antitumor effect of the selected analogue (ChBac3.4-1-COOH) point to the prospect of their future application in anticancer therapy, presumably in cases when the tumor grow is accompanied with a chronic infection, since the peptide exerts the dual antibacterial and antitumor action. We have shown that *C*-terminal amidation does not significantly affect the biological activity of the peptides and the recombinantly produced peptide possess the same potential as its chemically synthesized variants certifying that these AMPs can be produced using a more cost effective technology. Closer investigation of the exact mechanism of the antitumor action of ChBac3.4 is required to rationally design analogues with the improved activity, and further study is needed to assess the pharmacokinetics and pharmacodynamics of the peptides. In any case, the results obtained so far demonstrate the potential of structural modification to manage caprine bactenecins’ selectivity and activity spectrum and confirm that they are promising prototypes for antimicrobial and anticancer drugs development.

## Data Availability Statement

The raw data supporting the conclusions of this article will be made available by the authors, without undue reservation.

## Ethics Statement

The studies involving human participants were reviewed and approved by Local Ethics Committee at the Institute of Experimental Medicine, St-Petersburg. The patients/participants provided their written informed consent to participate in this study. The animal study was reviewed and approved by Local Ethics Committee at the Institute of Experimental Medicine, St-Petersburg.

## Author Contributions

OS, AT, and PK contributed conception and design of the study and participated in writing of the first draft of the manuscript, its reviewing ang editing. MZ performed antimicrobial testing experiments and the statistical analysis of the obtained data, and wrote the first draft of the manuscript. AAK, MPS, and ASK carried out the chemical synthesis and purification of the peptides. BM performed mass spectrometric analysis. MSS examined the hemolytic activity of the studied substances and carried out MTT tests. DO was responsible for the experiments on the evaluation of the effects of AMPs on bacterial membrane permeabilization. EU and VK carried out the antitumor activity examination *in vivo*. TO, PP, and SB produced the recombinant non-amidated peptide ChBac3.4-1-COOH and contributed the conception and design of the study of the antitumor potential of the studied peptides. All authors contributed to the article and approved the submitted version.

## Funding

This work was supported by the Ministry of Science and Higher Education of the Russian Federation (АААА-А19-119021290120-8) and Russian Foundation for Basic Research (RFBR projects No 17-04-02177a, 18-315-00333). The part of this work was supported by the RFBR project No. 18-54-80026.

## Conflict of Interest

The authors declare that the research was conducted in the absence of any commercial or financial relationships that could be construed as a potential conflict of interest.
