# Supplementary material for: Caprine Bactenecins as Promising Tools for Developing New Antimicrobial and Antitumor Drugs
Source: Front Cell Infect Microbiol. 2020 Oct 19;10:552905. doi: 10.3389/fcimb.2020.552905 (PMC7604311; doi:10.3389/fcimb.2020.552905)
Supplement: Supplementary file 1 [file DataSheet_1.pdf]

Supplementary Material

Supplementary Table 1. Susceptibility profiles of tested clinical isolates of bacteria.

antibiotic	Gram-negative:				antibiotic	Gram-positive:
	E. coli ESBL 521/17	*A. baumannii* 7226/16	*P. aeruginosa* MDR 522/17	*K. pneumoniae* ESBL 344/17		*S. aureus* 1399/17
AMP*	R [≥32]	R [≥32]	R [≥32]	R [≥32]	AMP*	R [≥32]
AMC	R [16]	-	-	S [≤2]	AMC	-
CTX	R [≥64]	-	-	S [≤1]	AMX	-
CAZ	R [≥64]	-	R [≥64]	S [≤1]	AZM	R [≥4]
FEP	R [≥64]	-	R [≥64]	S [≤1]	VAN	S [≤2]
ATM	R [≥64]	-	-	S [≤1]	DOX	I [2]
IPM	S [≤1]	R [≥16]	I [8]	S [≤1]	CLI	I [2]
MEM	S [≤0.25]	R [≥32]	R [≥16]	S [≤0.25]	MEM	I [4]
AMK	I [16]	I [8]	I [4]	S [≤2]	AMK	R [≥64]
GEN	S [≤1]	R [≥16]	R [≥16]	S [≤1]	GEN	R [≥32]
NET	R [8]	-	R [≥32]	S [≤1]	CEX	-
CIP	R [≥4]	-	R [≥4]	S [≤0.25]	CIP	S [≤1]
FOF	S [≤16]	-	-	S [≤16]	OFL	R [≥32]
NIT	R [128]	-	-	-	LVX	S [≤1]
CST	S [≤0.5]	-	R [≥16]	S [≤0.5]	CXM	-
SXT	R [≥320]	R [≥320]	-	S [≤20]	CRO	-
ERY	R [≥32]	R [≥32]	R [≥32]	R [≥32]	ERY	R [≥32]
TGC	-	I [4]	-	-	RXM	I [2]
SAM	-	I [16]	-	-	OX	R [≥32]

AMP* – ampicillin; AMC – amoxicillin–clavulanic acid; CTX – cefotaxime; CAZ – ceftazidime; FEP – cefepime; IPM – imipenem; MEM – meropenem; AMK – amikacin; GEN – gentamicin; CIP – ciprofloxacin; FOF – fosfomycin; NIT – nitrofurantoin; CST – colistin; SXT – trimethoprim–sulfamethoxazole; ATM – aztreonam; NET – netilmicin; SAM – ampicillin-sulbactam; TGC – tigecycline; ERY – erythromycin; AZM – azithromycin; AMX – amoxicillin; VAN – vancomycin; DOX – doxycycline; CLI – clindamycin; RXM – roxithromycin; CEX – cephalexin; OFL – ofloxacin; LVX – levofloxacin; CXM – cefuroxime; CRO – ceftriaxone; OX – oxacillin.
R – resistant; I – intermediate; S – susceptible (corresponding MIC values (mg/L) are given in parentheses).

Supplementary Table 2. Antimicrobial activity of ChBac3.4-1-COOH peptide obtained via the Fmoc solid phase synthesis (syn) or via recombinant expression in *E. coli* (rec) against Gram-positive and Gram-negative bacteria.

Peptides	MIC[a] (µM) against bacteria								GMIC[b]
	Gram-negative:					Gram-positive:			
	E. coli ML-35p	*A. baumannii* 7226/16	*K. pneumoniae* ESBL 344/17	*P. aeruginosa* MDR 522/17	*E. coli* ESBL 521/17	*S. aureus* SG-511	*MRSA* ATCC 33591	*S. aureus* 1399/17	
ChBac3.4-1-COOH (syn)	2-4	4	8	16	4-8	0.25	16	8	***4.8***
ChBac3.4-1-COOH (rec)	4	4	8	16	4-8	0.25	16	8	***5.0***

[a] Minimal inhibitory concentrations (MIC) are shown as medians of 3–6 independent experiments made in triplicates.
[b] Geometric mean of MICs measured against different bacterial strains (GMIC) was calculated for overall assessment of antimicrobial activity of tested ChBac3.4 variants.

Supplementary Table 3. Cytotoxic action of ChBac3.4-1-COOH peptide obtained via the Fmoc solid phase synthesis (syn) or via recombinant expression in *E. coli* (rec) toward normal or tumor eukaryotic cells and its selectivity indices (SI).

Peptides	IC_{50}[a] (µM) of cytotoxic action toward			$SI_{h/b}$[b]	$SI_{n/t}$[c]
	normal cells:	tumor cells:		$\frac{IC_{50}^{PBMC}}{GMIC}$ & [its ratio to ChBac3.4's one]	$\frac{IC_{50}^{PBMC}}{\sqrt{IC_{50}^{K562}\cdot IC_{50}^{U937}}}$ & [its ratio to ChBac3.4's one]
	human PBMC	K562	U937		
ChBac3.4-1-COOH (syn)	33.7 ± 3.2	16.5 ± 1.3	9.8 ± 0.5	7.0 [1.7]	2.7 [1.6]
ChBac3.4-1-COOH (rec)	40.8 ± 9.1	15.6 ± 4.8	9.7 ± 0.3	8.1 [2.0]	3.3 [1.9]

PBMC – peripheral blood mononuclear cells; K562 – human erythroleukemia cells; U937 – human histiocytic lymphoma cells.
[a] Half maximal inhibitory concentrations (IC_{50}) of cytotoxic action were calculated using Sigma Plot Standard Curve Analysis based on data of 3 independent MTT-tests and are shown as mean ± standard deviation. Mann–Whitney U-test ($p < 0.05$) revealed no significant difference between synthetic and recombinant ChBac3.4-1-COOH variants.
[b] Selectivity index illustrating how much the peptide "preferes" to damage bacterial cells over normal human cells ($SI_{h/b}$) was determined according to the formula specified in the table's header. In this particular case we divided IC_{50} towards human PBMC by the geometric mean of minimal inhibitory concentrations measured against different bacterial strains (GMIC, see Table 2) that we considered an overall assessment of antimicrobial activity. If more types of nontumor cells had been tested, we would have used the geometric mean of corresponding ICs_{50} as a numerator similarly to GMIC in denominator.
[c] Selectivity index demonstrating if toxic effects of the peptide toward tumor cells are higher than toward normal eukaryotic cells ($SI_{n/t}$) was calculated according to the formula given in the table's header. In general case it would have been a quotient of geometric mean of ICs_{50} toward all tested types of nontumor cells and geometric mean of ICs_{50} toward all tested types of tumor cells.
To assess the improvement in either types of selectivity the ratios of the corresponding SI of the peptide of interest to the corresponding SI of the native ChBac3.4-NH_2 were calculated; they are shown in square brackets.

Data on the synthetic peptides

<u>HPLC</u>

Control Method Notes:
Column: Luna C-18 250x4.6 mm, 5 µm; Mobile Phase: 0.1% TFA in water / Acetonitrile (Gradient: 7-70 % ACN for 20 min): Flow Rate: 1 ml/min; Temp. – 35°C; Detector: UV 220 nm

Operation List Notes:

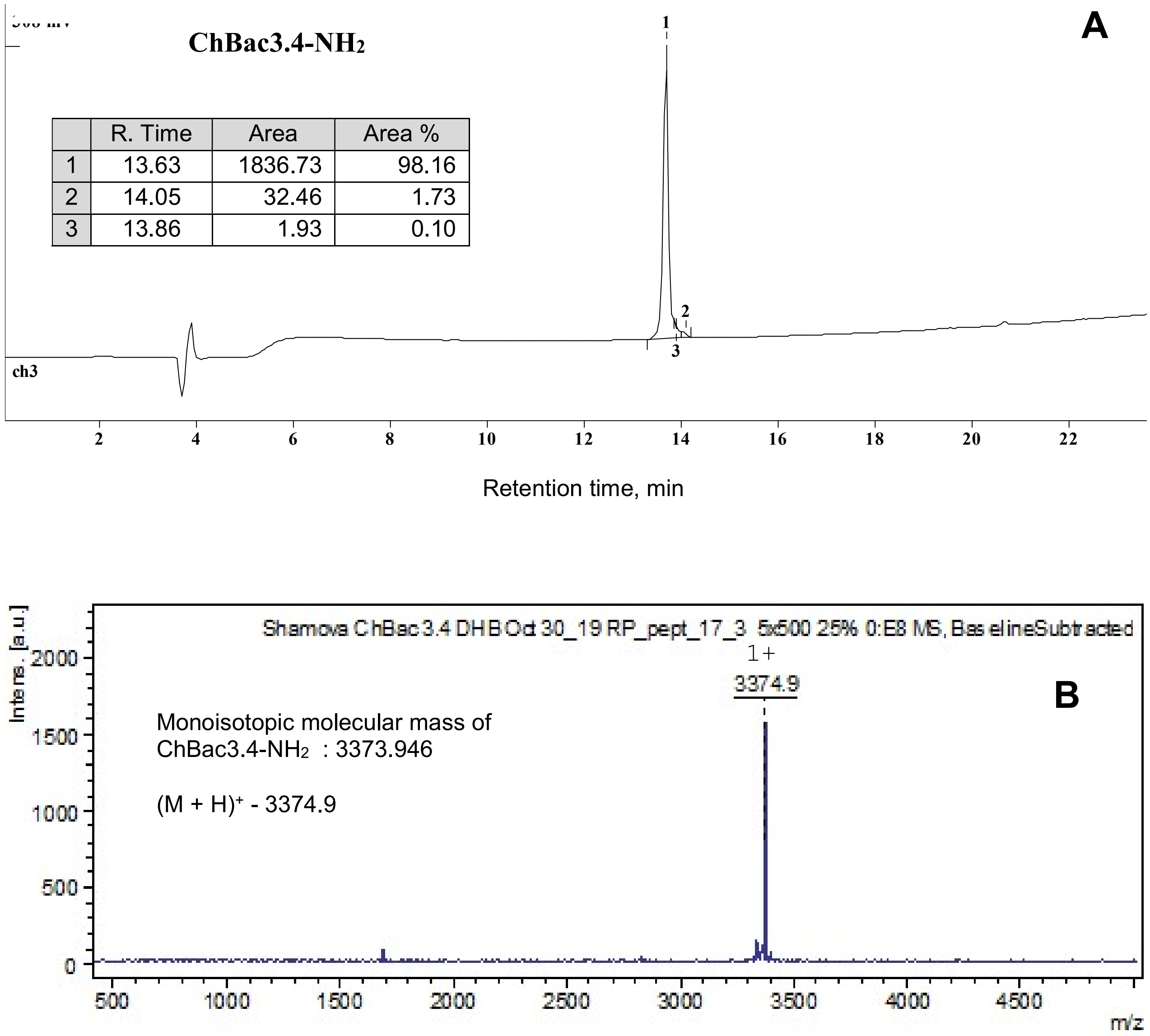

	R. Time	Area	Area %
1	13.63	1836.73	98.16
2	14.05	32.46	1.73
3	13.86	1.93	0.10

Supplementary Figure 1. Analytical HPLC (A) and MALDI TOF mass-spectrometry (B) data for the parent peptide ChBac3.4-NH_2.

HPLC

Control Method Notes:

Column: Luna C-18 250x4.6 mm, 5 µm; Mobile Phase: 0.1% TFA in water / Acetonitrile (Gradient: 7-70 % ACN for 20 min): Flow Rate: 1 ml/min; Temp. – 35ºC; Detector: UV 220 nm

Operation List Notes:

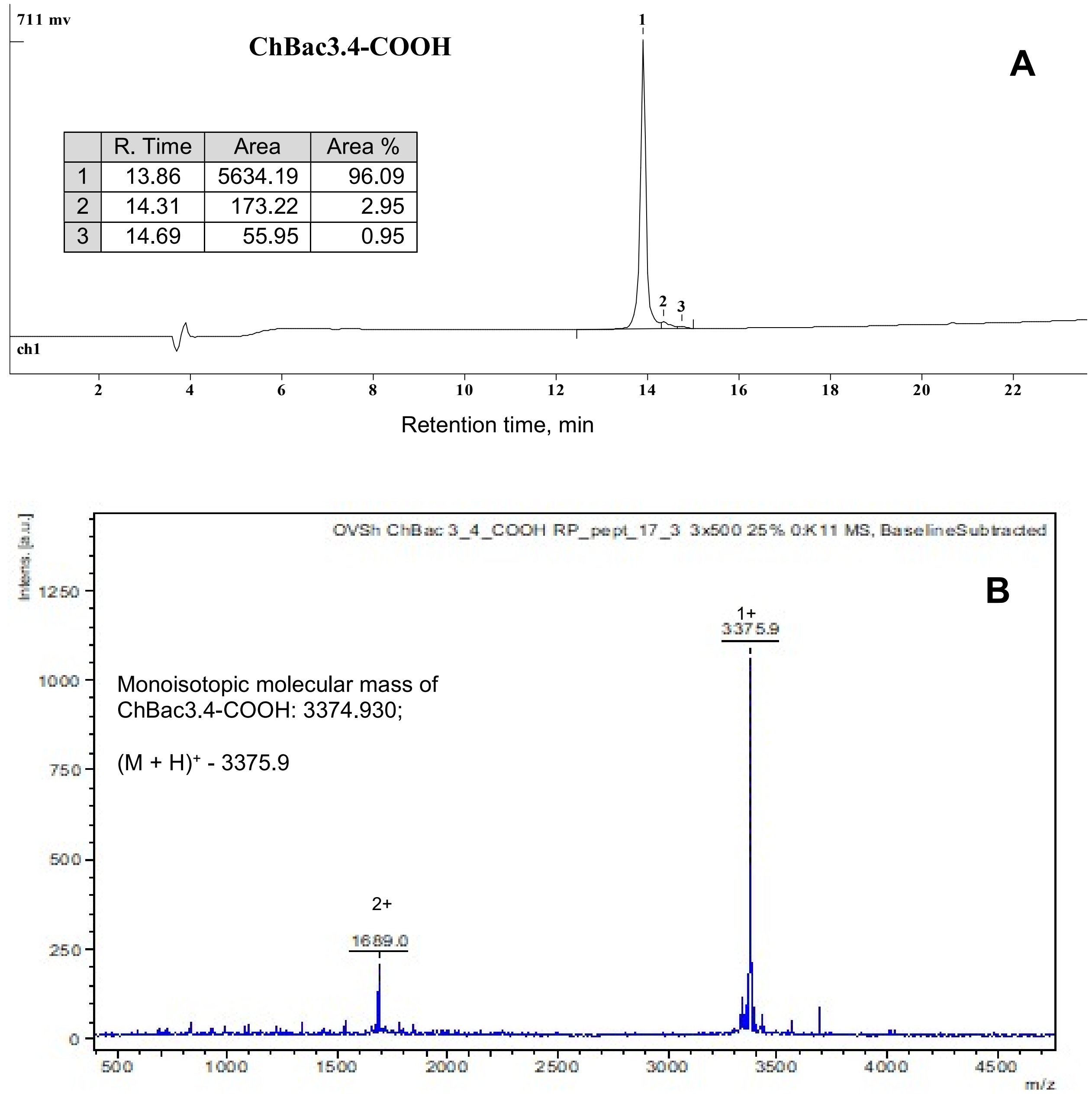

	R. Time	Area	Area %
1	13.86	5634.19	96.09
2	14.31	173.22	2.95
3	14.69	55.95	0.95

Supplementary Figure 2. Analytical HPLC (A) and MALDI TOF mass-spectrometry (B) data for ChBac3.4-COOH.

HPLC

Control Method Notes:

Column: Luna C-18 250x4.6 mm, 5 µm; Mobile Phase: 0.1% TFA in water / Acetonitrile (Gradient: 7-70 % ACN for 20 min): Flow Rate: 1 ml/min; Temp. – 35°C; Detector: UV 220 nm

Operation List Notes:

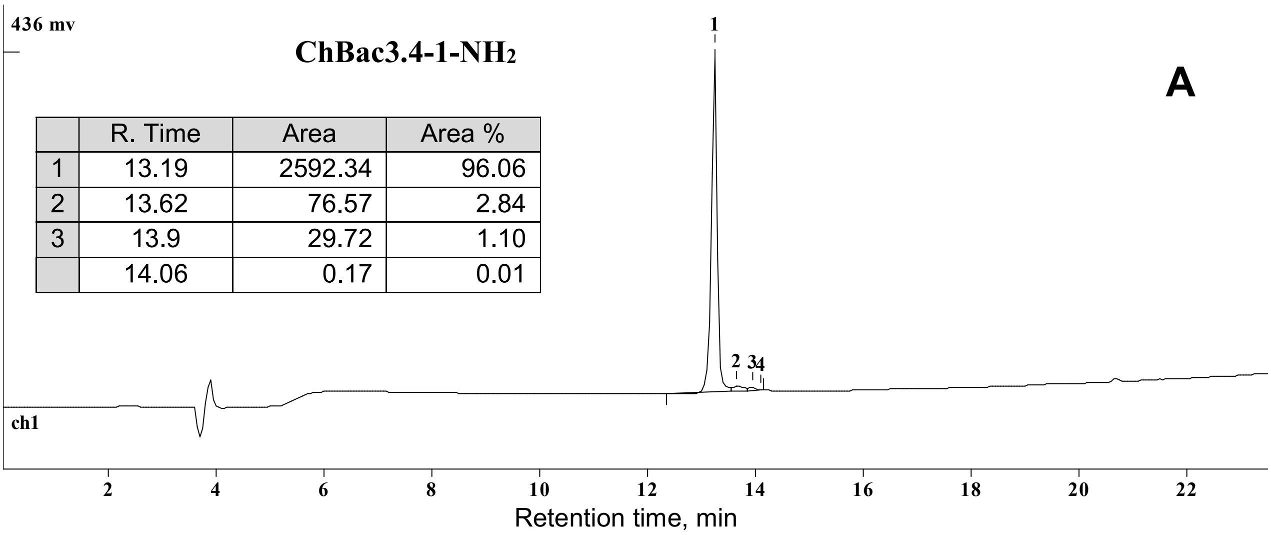

	R. Time	Area	Area %
1	13.19	2592.34	96.06
2	13.62	76.57	2.84
3	13.9	29.72	1.10
	14.06	0.17	0.01

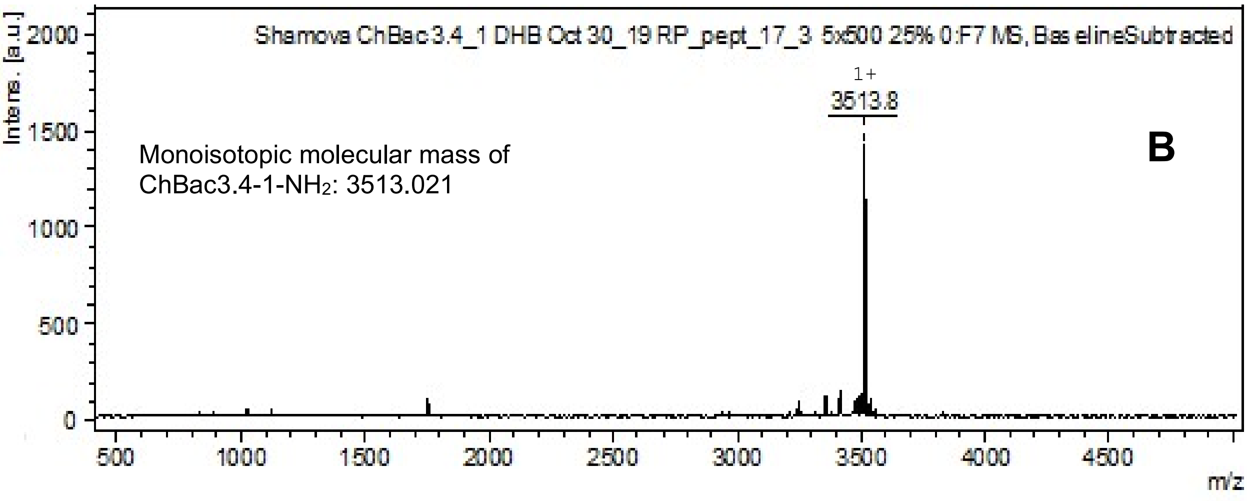

Supplementary Figure 3. Analytical HPLC (A) and MALDI TOF mass-spectrometry (B) data for ChBac3.4-1-NH_2.

HPLC

Control Method Notes:

Column: Luna C-18 250x4.6 mm, 5 µm; Mobile Phase: 0.1% TFA in water / Acetonitrile (Gradient: 7-70 % ACN for 20 min): Flow Rate: 1 ml/min; Temp. – 35°C; Detector: UV 220 nm

Operation List Notes:

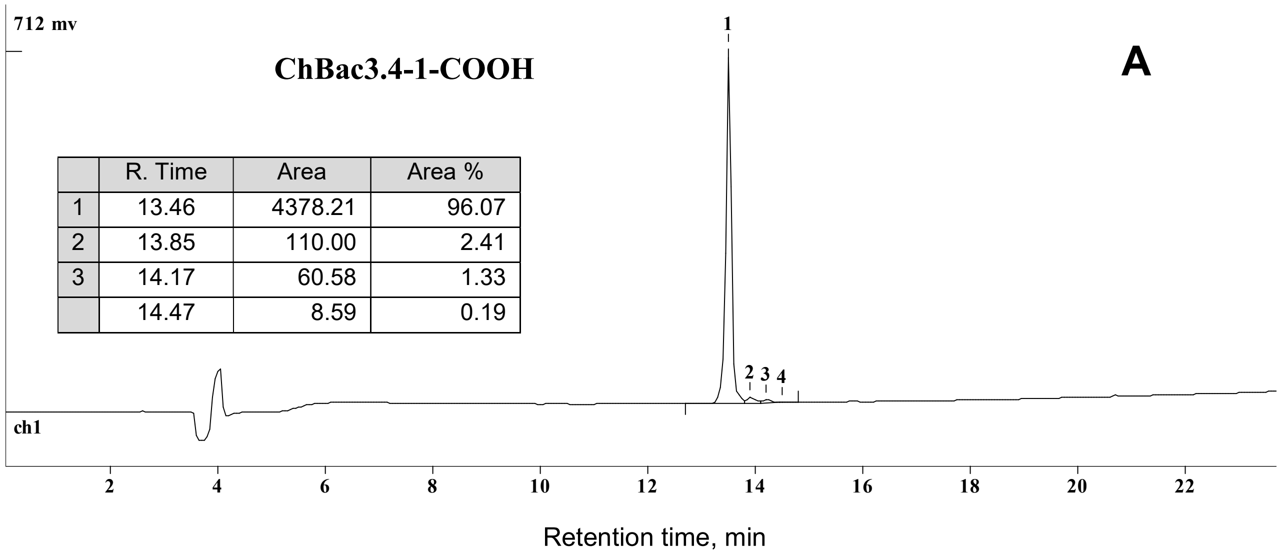

	R. Time	Area	Area %
1	13.46	4378.21	96.07
2	13.85	110.00	2.41
3	14.17	60.58	1.33
	14.47	8.59	0.19

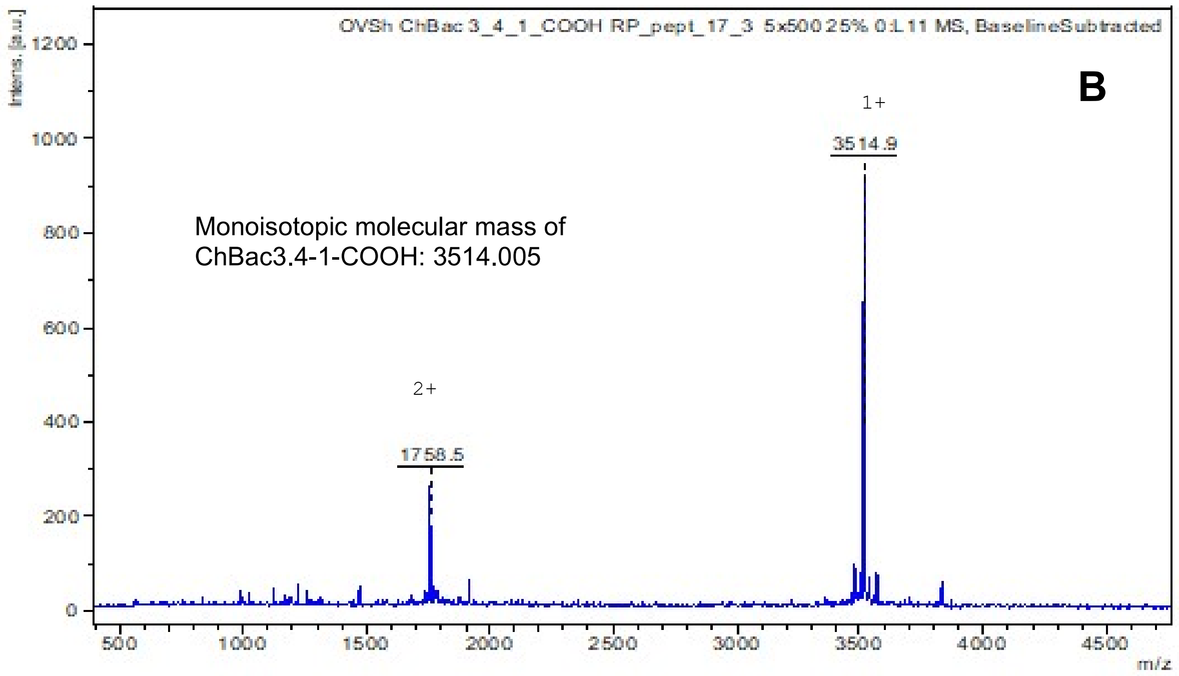

Supplementary Figure 4. Analytical HPLC (A) and MALDI TOF mass-spectrometry (B) data for ChBac3.4-1-COOH.

HPLC

Control Method Notes:

Column: Luna C-18 250x4.6 mm, 5 µm; Mobile Phase: 0.1% TFA in water / Acetonitrile (Gradient: 7-70 % ACN for 20 min): Flow Rate: 1 ml/min; Temp. – 35°C; Detector: UV 220 nm

Operation List Notes:

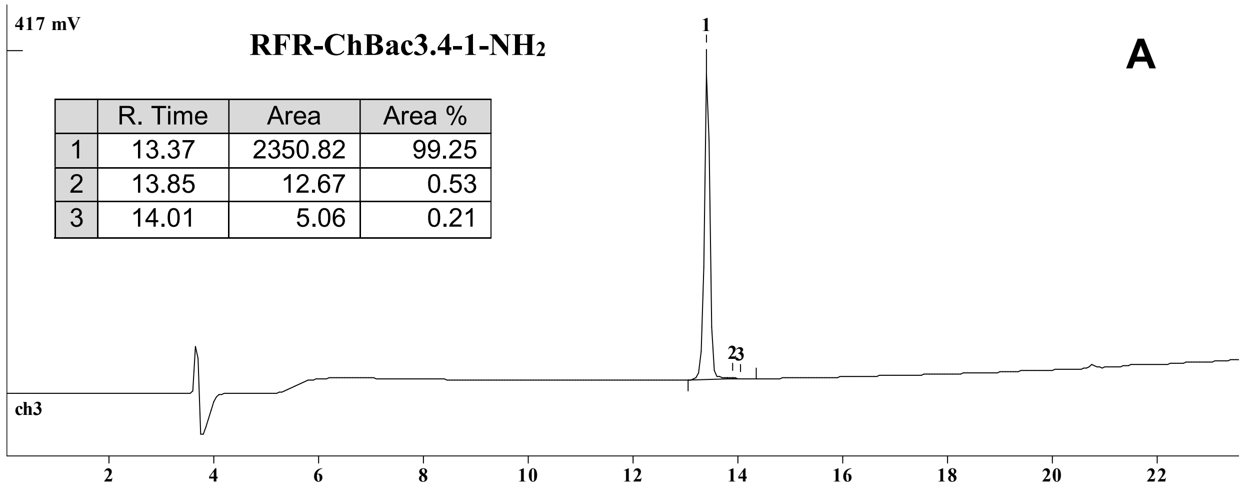

	R. Time	Area	Area %
1	13.37	2350.82	99.25
2	13.85	12.67	0.53
3	14.01	5.06	0.21

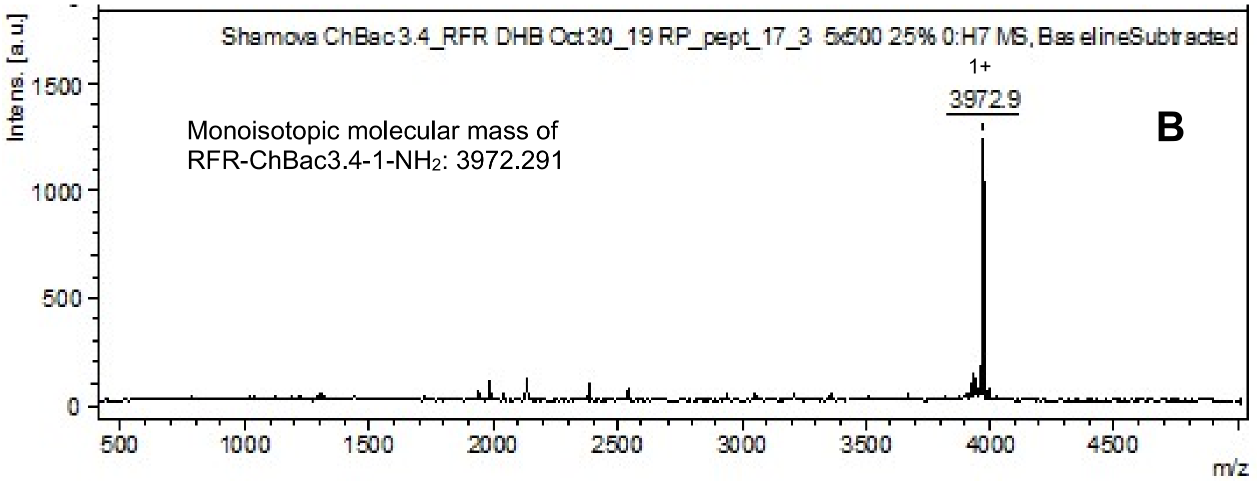

Supplementary Figure 5. Analytical HPLC (A) and MALDI TOF mass-spectrometry (B) data for RFR-ChBac3.4-1-NH_2.

HPLC

Control Method Notes:

Column: Luna C-18 250x4.6 mm, 5 µm; Mobile Phase: 0.1% TFA in water / Acetonitrile (Gradient: 7-70 % ACN for 20 min): Flow Rate: 1 ml/min; Temp. – 35ºC; Detector: UV 220 nm

Operation List Notes:

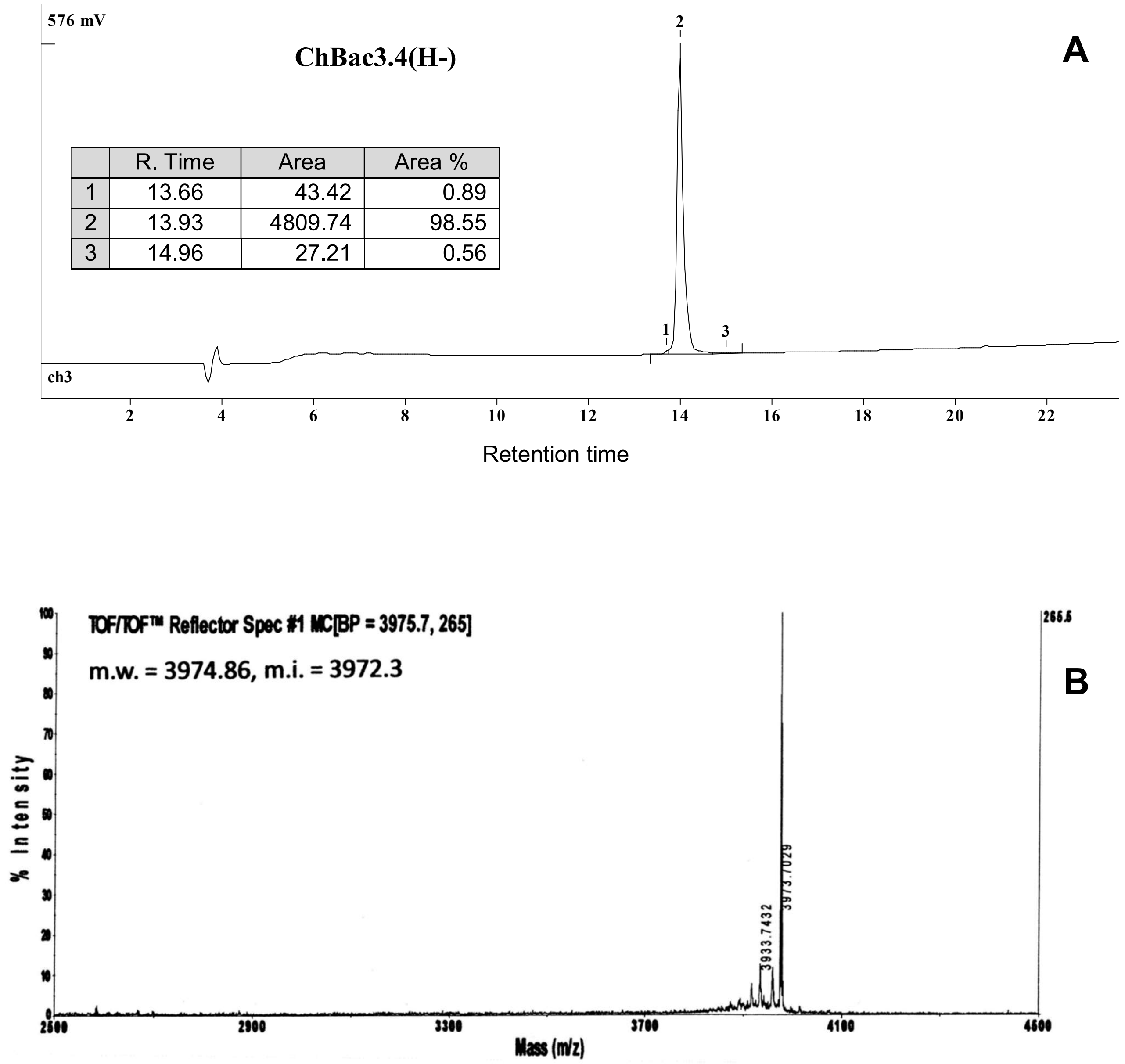

	R. Time	Area	Area %
1	13.66	43.42	0.89
2	13.93	4809.74	98.55
3	14.96	27.21	0.56

Supplementary Figure 6. Analytical HPLC (A) and MALDI TOF mass-spectrometry (B) data for ChBac3.4 (H-).

HPLC

Control Method Notes:

Column: Luna C-18 250x4.6 mm, 5 μm; Mobile Phase: 0.1% TFA in water / Acetonitrile (Gradient: 7-70 % ACN for 20 min): Flow Rate: 1 ml/min; Temp. – 35°C; Detector: UV 220 nm

Operation List Notes:

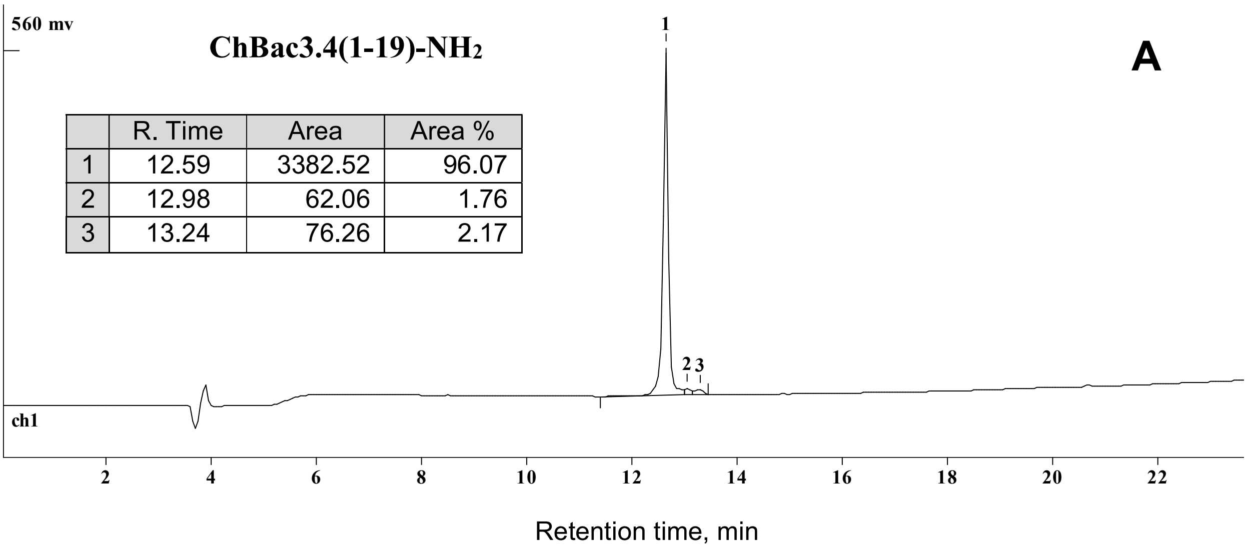

	R. Time	Area	Area %
1	12.59	3382.52	96.07
2	12.98	62.06	1.76
3	13.24	76.26	2.17

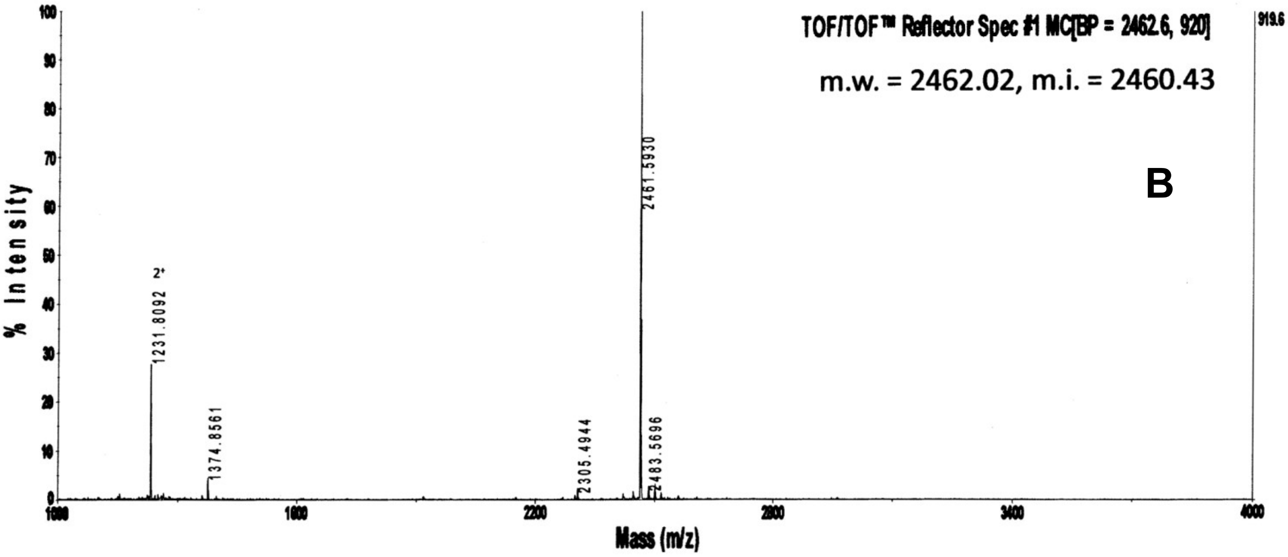

Supplementary Figure 7. Analytical HPLC (A) and MALDI TOF mass-spectrometry (B) data for ChBac3.4(1-19)-NH_2.

HPLC

Control Method Notes:

Column: Luna C-18 250x4.6 mm, 5 µm; Mobile Phase: 0.1% TFA in water / Acetonitrile (Gradient: 7-70 % ACN for 20 min): Flow Rate: 1 ml/min; Temp. – 35ºC; Detector: UV 220 nm

Operation List Notes:

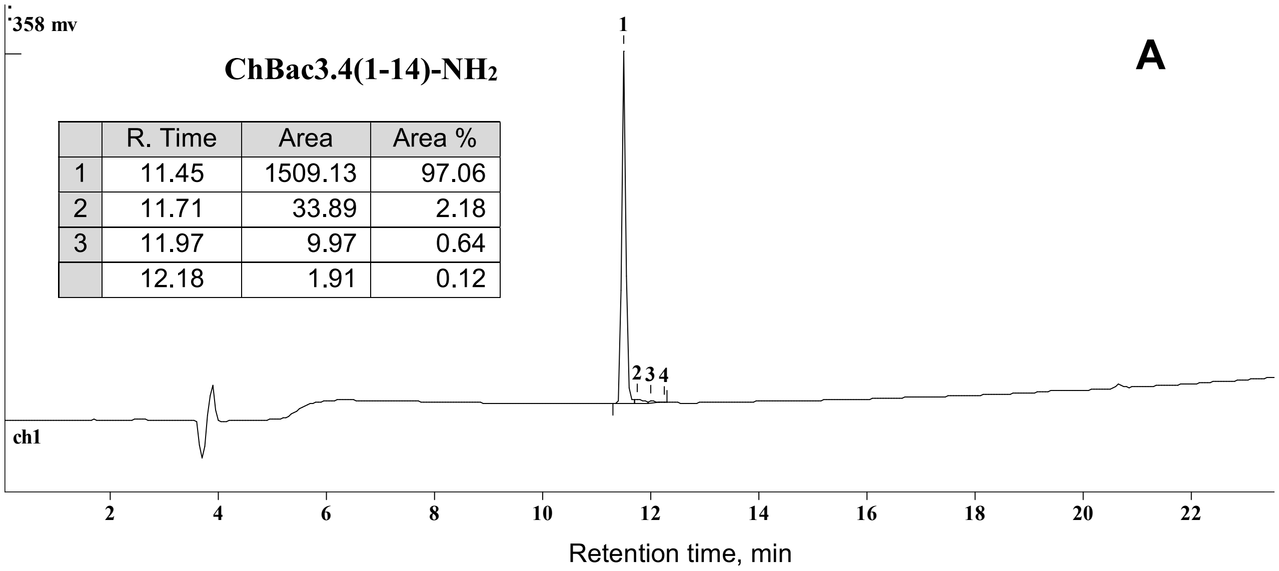

	R. Time	Area	Area %
1	11.45	1509.13	97.06
2	11.71	33.89	2.18
3	11.97	9.97	0.64
	12.18	1.91	0.12

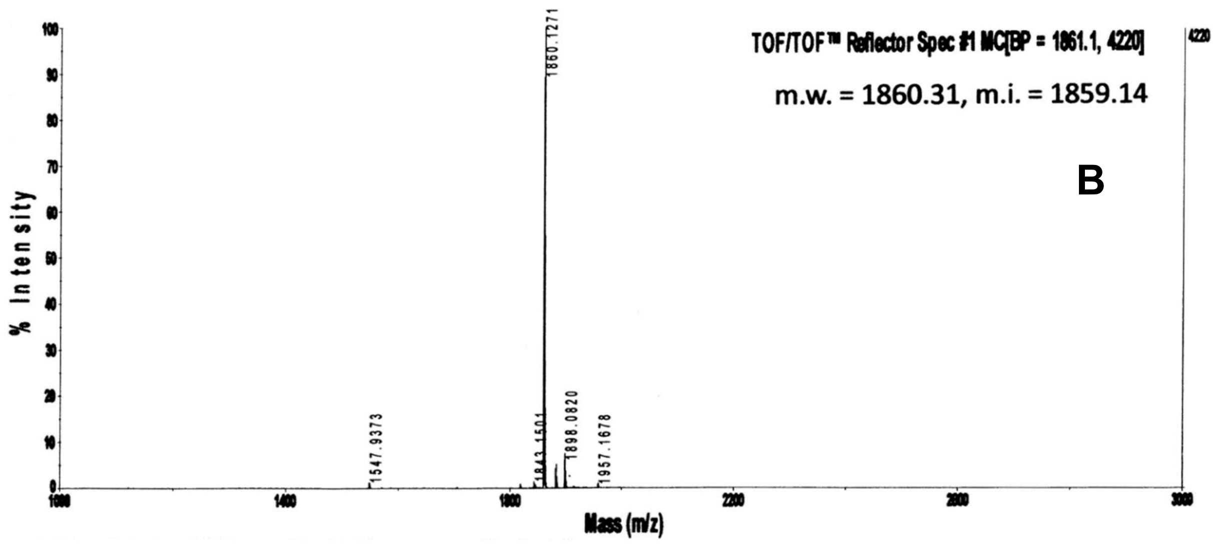

Supplementary Figure 8. Analytical HPLC (A) and MALDI TOF mass-spectrometry (B) data for ChBac3.4(1-14)-NH_2.

HPLC

Control Method Notes:

Column: Luna C-18 250x4.6 mm, 5 µm; Mobile Phase: 0.1% TFA in water / Acetonitrile (Gradient: 7-70 % ACN for 20 min): Flow Rate: 1 ml/min; Temp. – 35°C; Detector: UV 220 nm

Operation List Notes:

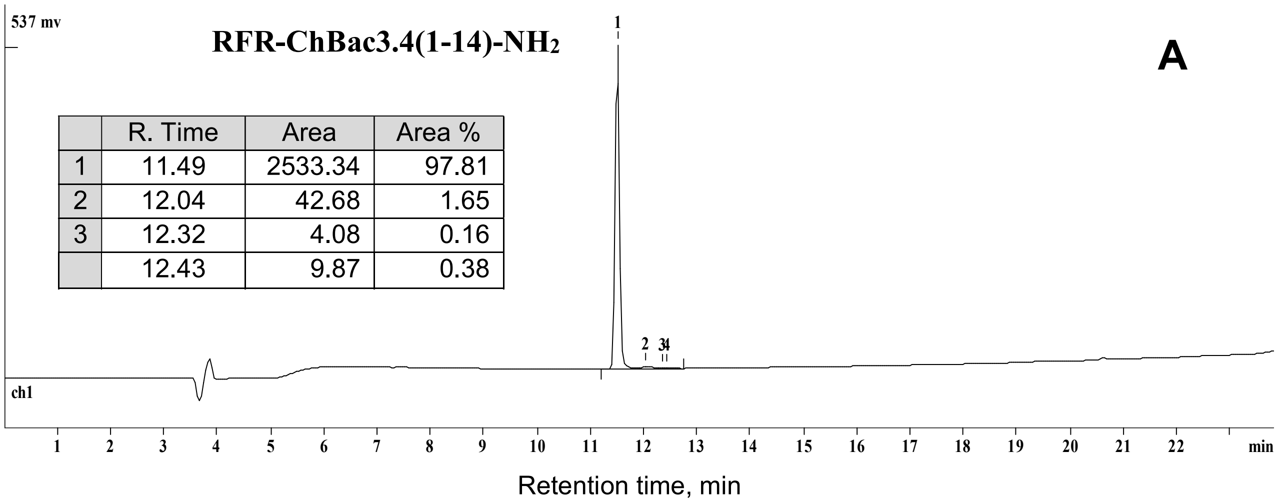

	R. Time	Area	Area %
1	11.49	2533.34	97.81
2	12.04	42.68	1.65
3	12.32	4.08	0.16
	12.43	9.87	0.38

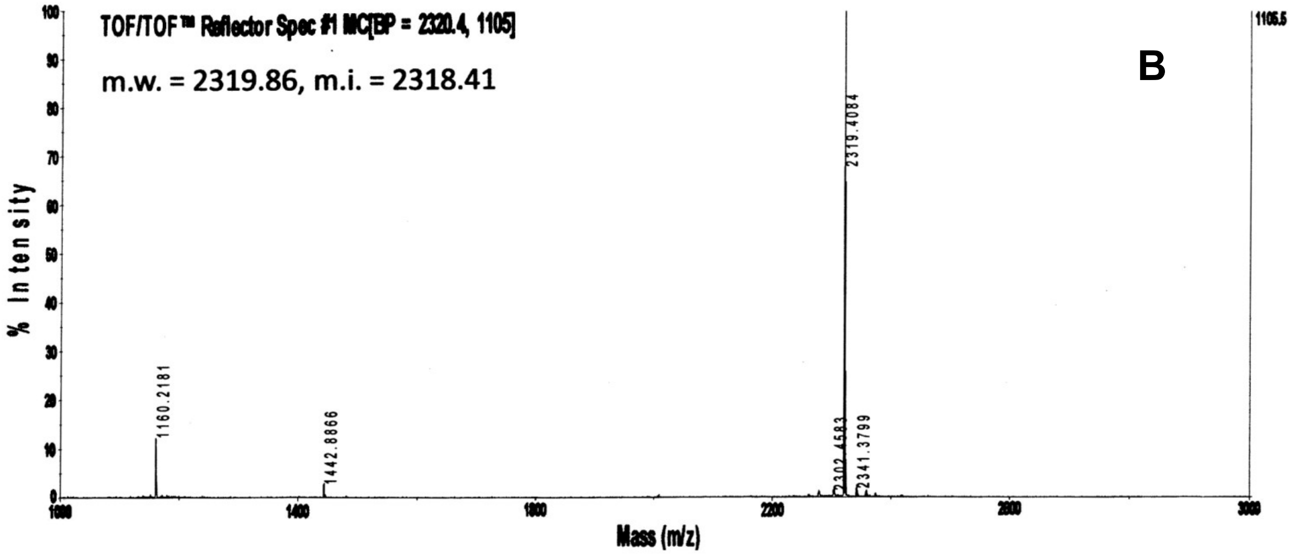

Supplementary Figure 9. Analytical HPLC (A) and MALDI TOF mass-spectrometry (B) data for RFR-ChBac3.4(1-14)-NH_2.

HPLC

Control Method Notes:

Column: Luna C-18 250x4.6 mm, 5 µm; Mobile Phase: 0.1% TFA in water / Acetonitrile (Gradient: 7-70 % ACN for 20 min): Flow Rate: 1 ml/min; Temp. – 35°C; Detector: UV 220 nm

Operation List Notes:

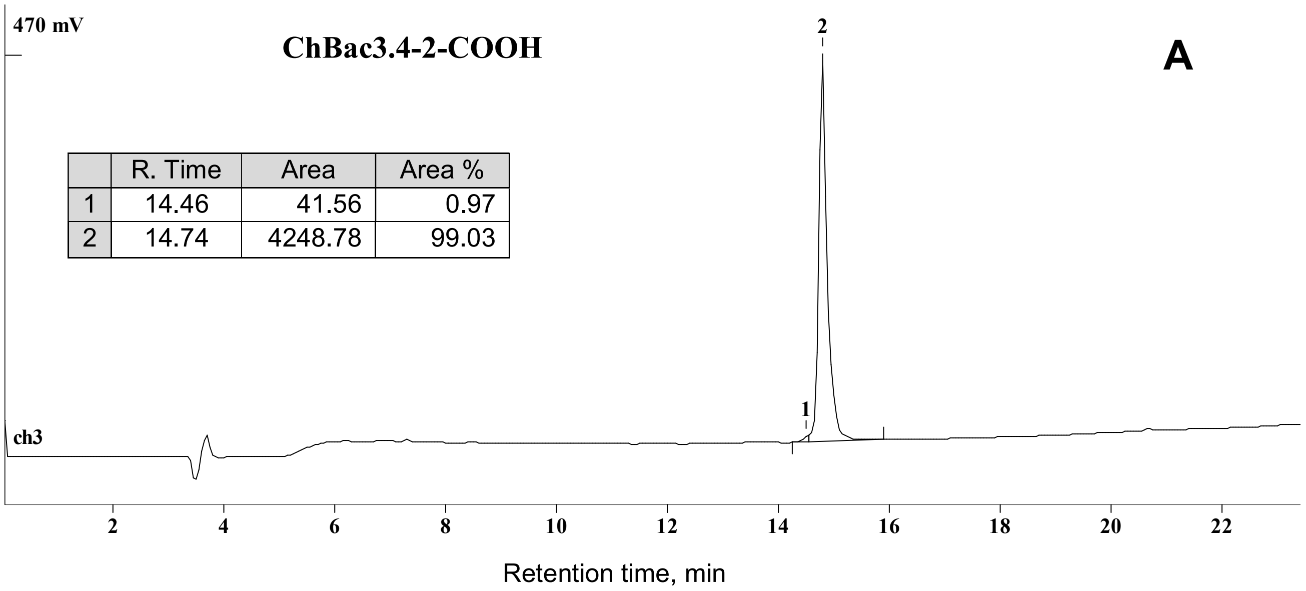

	R. Time	Area	Area %
1	14.46	41.56	0.97
2	14.74	4248.78	99.03

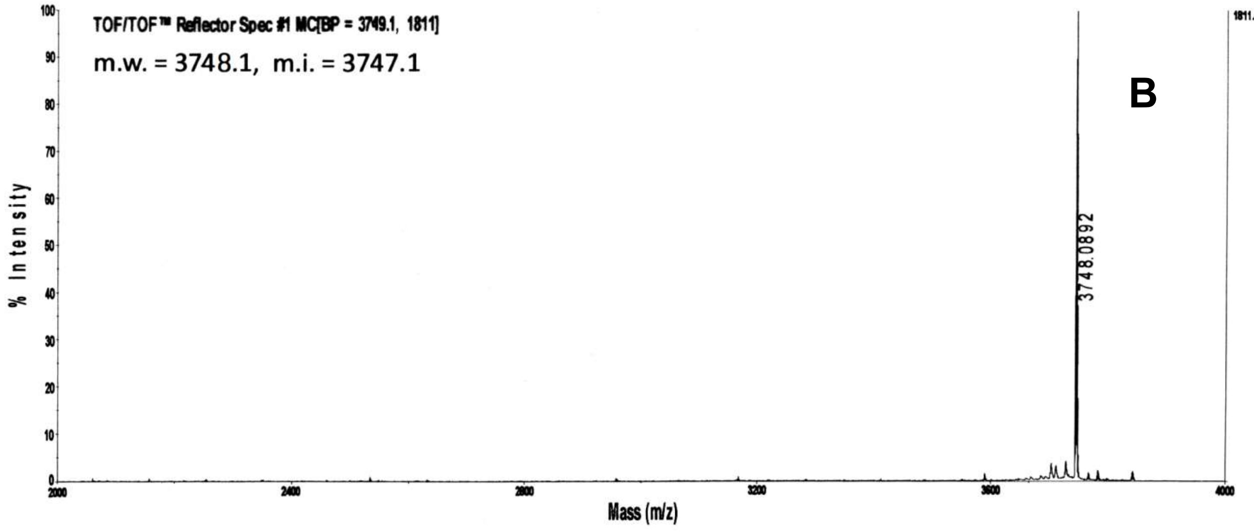

Supplementary Figure 10. Analytical HPLC (A) and MALDI TOF mass-spectrometry (B) data for ChBac3.4-2-COOH.

HPLC

Control Method Notes:

Column: Luna C-18 250x4.6 mm, 5 µm; Mobile Phase: 0.1% TFA in water / Acetonitrile (Gradient: 7-70 % ACN for 20 min): Flow Rate: 1 ml/min; Temp. – 35°C; Detector: UV 220 nm

Operation List Notes:

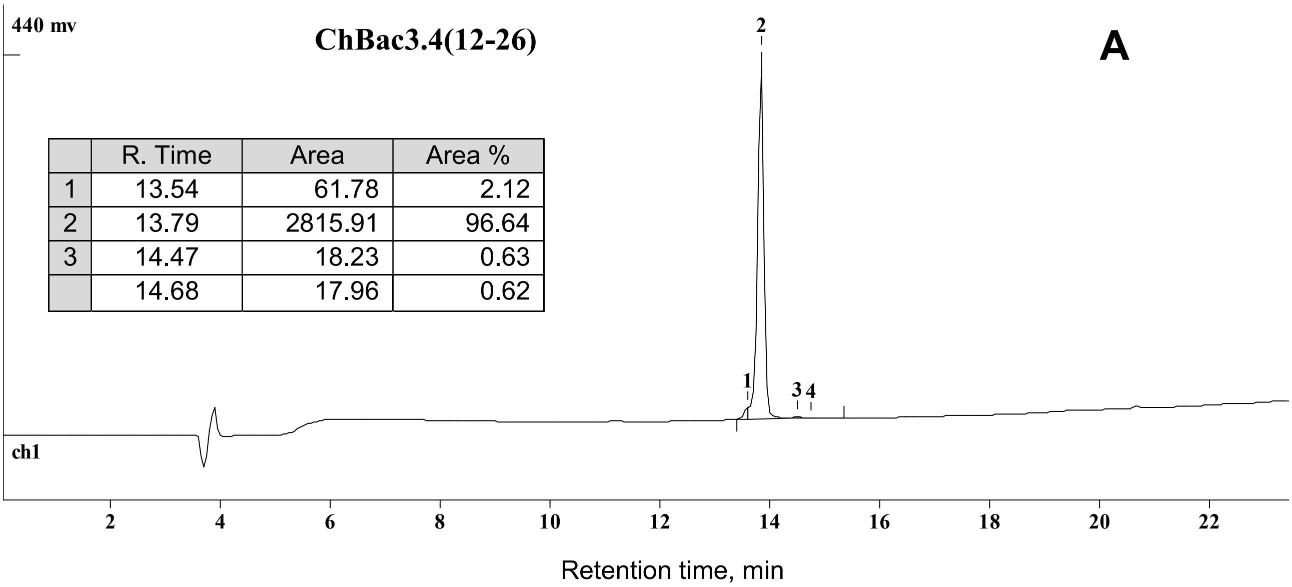

	R. Time	Area	Area %
1	13.54	61.78	2.12
2	13.79	2815.91	96.64
3	14.47	18.23	0.63
	14.68	17.96	0.62

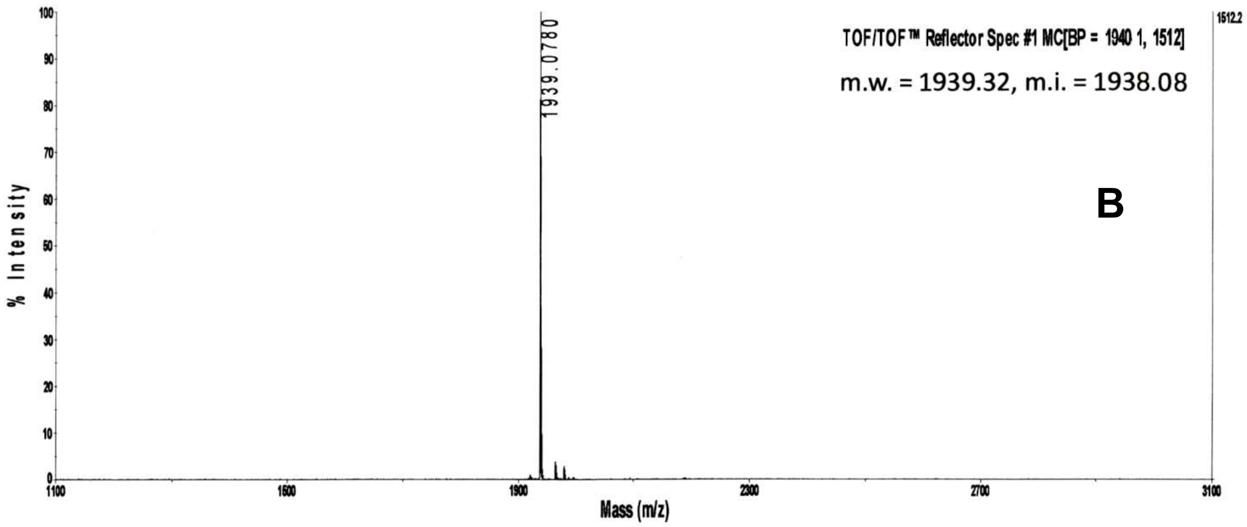

Supplementary Figure 11. Analytical HPLC (A) and MALDI TOF mass-spectrometry (B) data for ChBac3.4(12-26).

Data on the recombinant peptide ChBac3.4-1-COOH

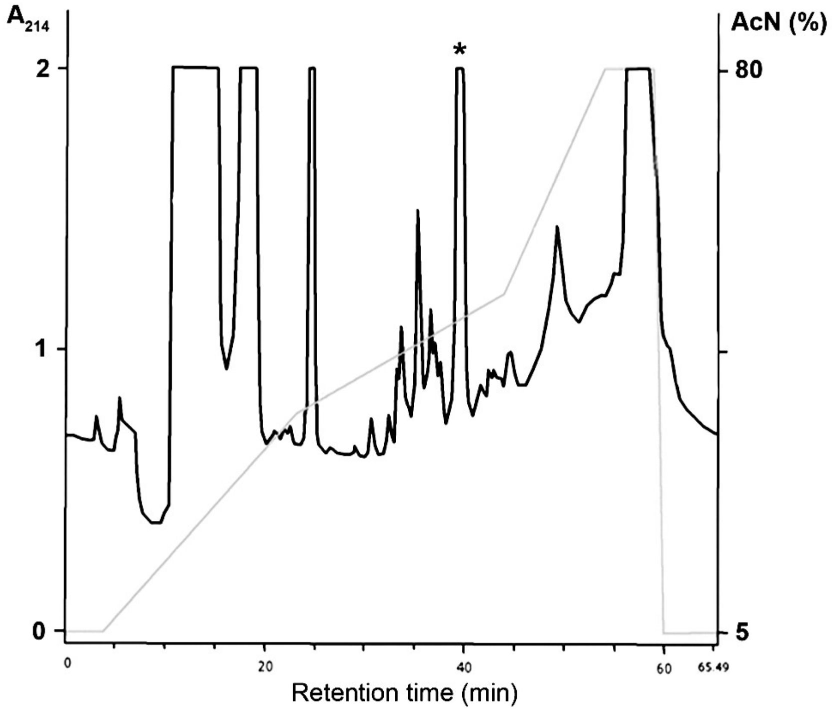

Supplementary Figure 12. Reversed-phase high-performance liquid chromatography (RP-HPLC) of the recombinant peptide ChBac3.4-1-COOH. RP-HPLC was performed with a gradient from 5 to 80% (v/v) of acetonitrile in water containing 0.1% TFA. The mature recombinant peptide fraction is marked with an asterisk.

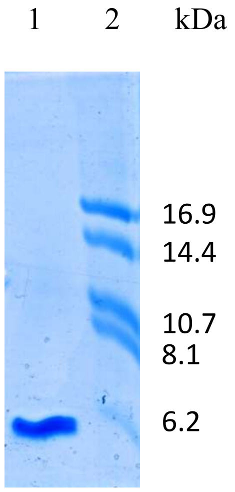

Supplementary Figure 13. SDS-PAGE of the purified recombinant peptide ChBac3.4-1-COOH. Lane 1 – ChBac 3.4-1-COOH; lane 2 – low molecular weight protein ladder.

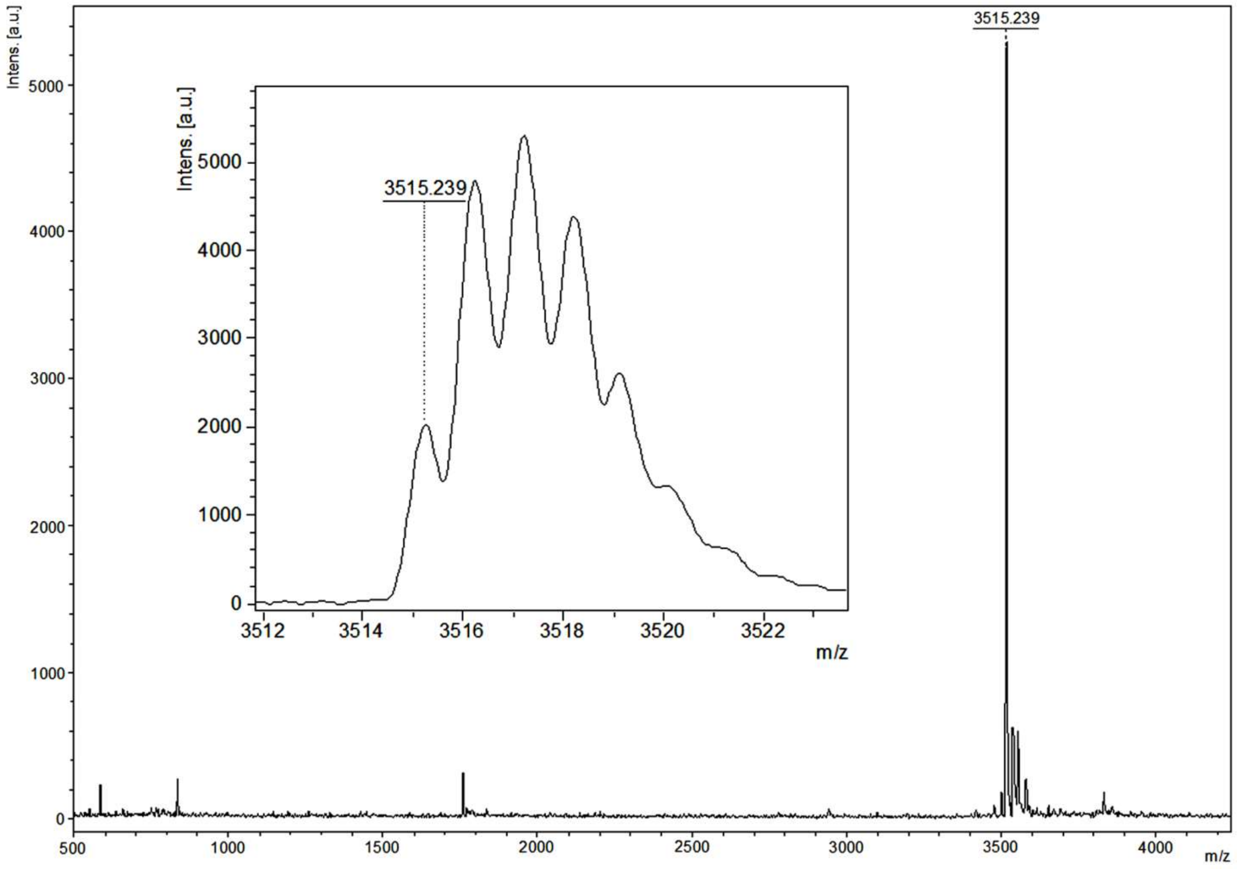

Supplementary Figure 14. MALDI-MS analysis of the recombinant peptide ChBac3.4-1-COOH. The measured monoisotopic m/z value of the peptide (3515.2) matched the corresponding calculated molecular mass of 3515.0 Da.
